# Maternal genetic features of the Iron Age Tagar population from Southern Siberia (1^st^ millennium BC)

**DOI:** 10.1371/journal.pone.0204062

**Published:** 2018-09-20

**Authors:** Aleksandr S. Pilipenko, Rostislav O. Trapezov, Stepan V. Cherdantsev, Vladimir N. Babenko, Marina S. Nesterova, Dmitri V. Pozdnyakov, Vyacheslav I. Molodin, Natalia V. Polosmak

**Affiliations:** 1 Institute of Cytology and Genetics, Siberian Branch, Russian Academy of Sciences, Novosibirsk, Russia; 2 Institute of Archaeology and Ethnography, Siberian Branch, Russian Academy of Sciences, Novosibirsk, Russia; 3 Novosibirsk State University, Novosibirsk, Russia; Universitat Pompeu Fabra, SPAIN

## Abstract

Early nomads in the Eurasian steppes since the beginning of the 1^st^ millennium BC played a key role in the formation of the cultural and genetic landscape of populations of a significant part of Eurasia, from Eastern Europe to Eastern Central Asia. Numerous archaeological cultures associated with early nomads have been discovered throughout the Eurasian steppe belt. The Tagar archaeological culture existed in the Minusinsk basin (Sayan Mountains, Southern Siberia, Russia) in the northeastern periphery of the Eurasian steppe belt from the 8^th^ to 1^st^ century BC during the pre-Scythian, Scythian, and Early Xiongnu-Sarmatian periods. In this study, we evaluated mtDNA diversity in the Tagar population based on representative series (N = 79) belonging to all chronological stages of the culture. The Tagar population had a mixed mtDNA pool dominated by Western Eurasian haplogroups and subgroups (H, HV6, HV*, I, K, T, U2e, U4, U5a, and U*) and, to a lesser degree, Eastern Eurasian haplogroups (A*, A8, C*, C5, D, G2a, and F1b). The Tagar population showed a similar mtDNA pool structure to those of other Iron Age populations representing the “Scythian World.” We observed particularly high similarity between the Tagar and Classic Scythians from the North Pontic region. Our results support the assumption that genetic components introduced by Bronze Age migrants from Western Eurasia contributed to the formation of the genetic composition of Scythian period populations in Southern Siberia. Another important component of the Tagar mtDNA pool was autochthonous East Eurasian lineages, some of which (A8 and C4a2a) are potential markers of the westward genetic influence of the eastern populations of the Scythian period. Our results suggest a genetic continuity (at least partial) between the Early, Middle, and Late Tagar populations.

## Introduction

In the 1^st^ millennium BC, numerous groups of early nomads arose and spread within the steppe belt of Eurasia. Early nomadic groups were the driving force of large-scale demographic events in Eurasia during the Early Iron Age. The range of early nomads encompasses the Eurasian steppes from the North Pontic region in the west to Southern Siberia and Eastern Central Asia in the east. Several stages in the development of these early nomadic communities, such as pre-Scythian, Scythian, and Xiongnu-Sarmatian periods, are distinguished based on archaeological and historical data. A characteristic feature of these periods is the widespread distribution of common elements in material cultures within the steppe belt and adjacent territories, accompanied by the intensification of cultural and, apparently, genetic interactions among populations from geographically remote regions of Eurasia.

Numerous local groups of early nomads have been studied using methods in archaeology and physical anthropology. These studies have elucidated the local features of the material culture of early nomads from different regions of the Eurasian steppe and have enabled the reconstruction of probable internal and external cultural vectors and, to some extent, genetic links. Large collections of skeletal remains have accumulated, many of which have been characterized by methods in physical anthropology (craniometry, odontology, etc.). These paleoanthropological collections have been used for molecular genetic studies of the Early Iron Age nomads, from single samples and small local series [1–3] to comparative studies of the genetic composition of nomadic populations from geographically remote steppe regions, using both uniparental markers, especially mitochondrial DNA, and whole nuclear genome data [4].

The Tagar culture is one of the most archaeologically well-studied groups of early nomads from Southern Siberia. The Tagar archaeological culture existed in the territory of the Minusinsk basin (the Sayan mountain system, middle reaches of the Yenisei River, Republic of Khakassia, Russia) in the northeastern periphery of the Eurasian steppe belt for almost the entire 1^st^ millennium BC, that is, during the pre-Scythian, Scythian, and Early Xiongnu-Sarmatian periods. During this time, the Tagar culture progressed through several stages (for details of these stages see S4 File), which differ in material culture features and funeral practices [5, 6]. According to a generally accepted archaeological hypothesis, stages of the Tagar culture shared a common cultural basis [5, 6]. But several waves of external cultural influence could be a driving force for changes in the Tagar material culture and burial practice [6].

To date, several thousand burials and numerous other sites belonging to the Tagar culture have been excavated in the Minusinsk basin and adjacent territories (for a review of the excavated Tagar sites, see [5, 7]). Despite the accumulation of large amounts of archaeological and anthropological materials, many issues related to the Tagar culture remain unresolved, especially with respect to the biological (genetic) characteristics of Tagar populations. The genetic roots of the Tagar population, the degree of genetic continuity during different chronological stages of the Tagar culture (Early, Middle, and Late stages), and interactions with other groups of early nomads from adjacent regions of Southern Siberia, Central Asia, and other regions of Eurasia remain unclear.

There are uncertainties related to the formation of the Tagar population. The Minusinsk basin was not isolated from external migration flows, which were especially intense during the Bronze Age. The degree of influence of Bronze Age migrants and autochthonous genetic components in the initial formation of the Tagar population and external influences at different stages of the culture are subjects of discussion among archaeologists [6, 8, 9]. Cultural and genetic contacts with synchronous groups of early nomads from other regions of Southern Siberia are also unclear. The relative originality of Tagar material culture with some clearly similar features with surrounding Early Iron Age populations (including epochal features) is archaeologically noted [6].

Despite large collections of Tagar paleoanthropological materials, paleogenetic studies are limited. The paleogenetic data published to date include small series of mtDNA and Y-chromosome samples without a specified chronology (only the chronological frames of the entire culture are indicated) [10] and several mtDNA samples from the Barsuchiy Log site belonging to the Middle stage of the Tagar culture [4], for a total of 16 mtDNA samples (S3 File). Thus, previously published data are insufficient to characterize the genetic makeup of the Tagar population as a whole or to assess the genetic dynamics of the Tagar population and its genetic links with other ancient populations of Eurasia.

In this study, we evaluated mtDNA diversity in the Tagar population using a representative series (79 individuals) from burials belonging to all major chronological stages of the Tagar culture. We discuss the results of genetic analyses in the context of the issues of the Tagar population origin, its relationship with other ancient populations in Southern Siberia and other regions, and the dynamics of its genetic structure during the 1^st^ millennium BC.

## Materials and methods

### Paleoanthropological materials

**Fig 1:**
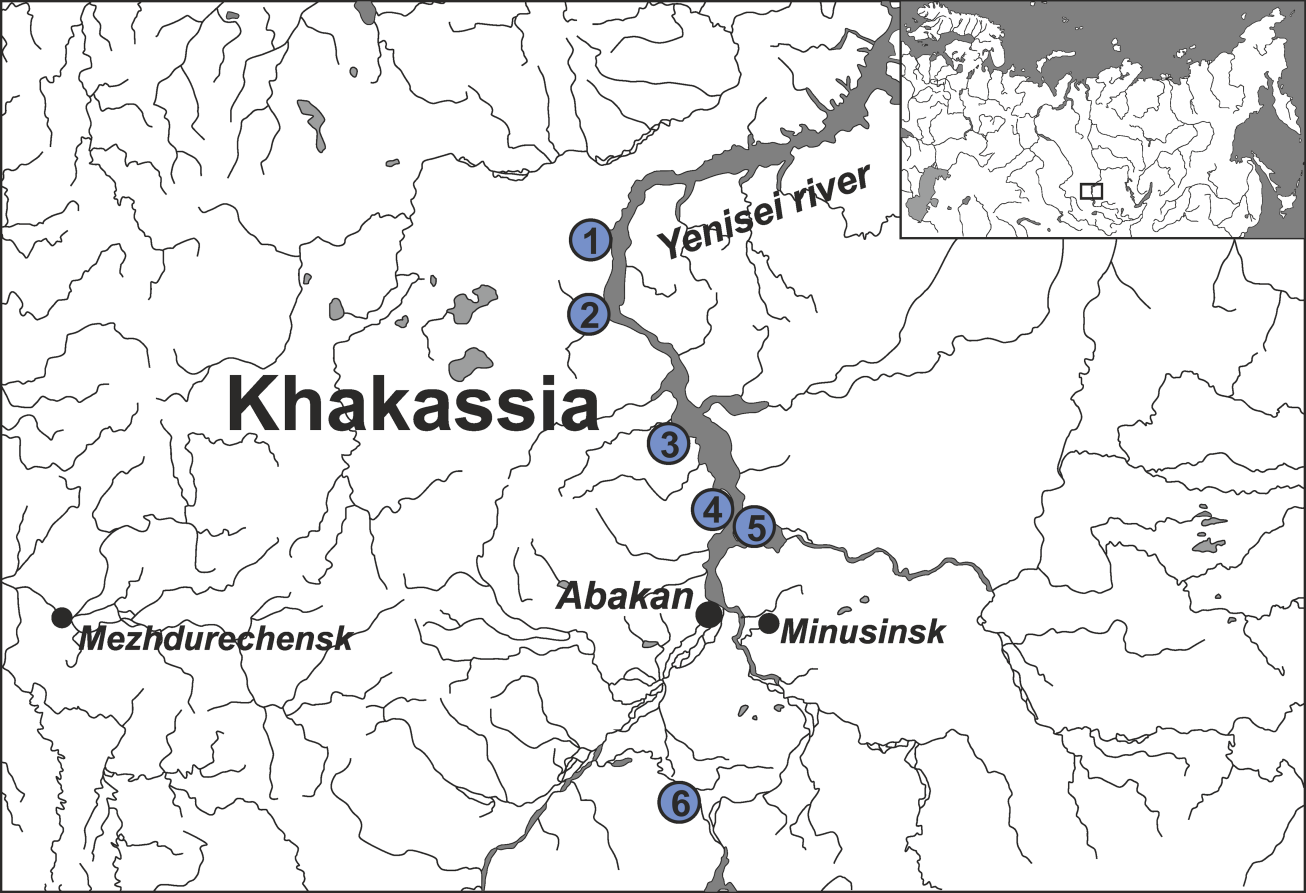
Location of Tagar archaeological sites from which samples for this study were obtained. Burial grounds: 1—Novaya Chernaya-1; 2—Podgornoe Ozero, Barsuchiha-1, Barsuchiha-6, Barsuchiha-7; 3—Perevozinskiy; 4—Ulug-Kyuzyur, Kichik-Kyuzyur, Sovetskaya Khakassiya; 5—Tepsey-3, Tepsey-8, Tepsey-9; 6—Dolgiy Kurgan.

The main area of the Tagar culture covers the steppe and forest-steppe zone in the basin of the middle Yenisei River and its tributaries (mainly in modern Khakassia Republic, Russia). Paleoanthropological materials from 95 individuals from 13 burial grounds of the Tagar culture located on the territory of the Minusinsk basin in the central part of the Tagar culture area were obtained (see details on Fig 1, S1 File). Materials from funeral complexes related to the main stages of the development of the Tagar culture, which replaced each other and were distinguished on the basis of archaeological data, are represented, with a predominance of materials from the Early and Middle stages: 46 samples (individuals) belonged to the Early Tagar period (Bainovo, Podgornovo, and Bidjinski types, according to the classification of M.P. Gryaznov) dated 9–6 centuries BC, 24 samples (individuals) belonged to the Middle Tagar period (Saragash type) dated 5–3 centuries BC, and 9 samples (individuals) belonged to the Late Tagar (Tes`type or Tes`culture) dated 2–1 centuries BC (see S1 File for detailed description of samples).

Most of paleoanthropological materials from the Tagar population studied in this work were obtained during excavations conducted in the Minusinsk Basin by the Krasnoyarsk Archaeological Expedition of the Leningrad Division of the Institute of Archeology of the USSR Academy of Sciences in 1955–70 (head of the expedition—M.P. Gryaznov) [8]).

All specimens analyzed in this study were obtained from Repository of Paleoanthropological Collections of the Institute of Archaeology and Ethnography, Siberian Branch, Russian Academy of Sciences (IAET SB RAS Novosibirsk, Russia). All materials are available for further studies according to the full description (including the name of archaeological site (burial ground name), number of burial (and skeleton number for double or collective burials)) given in S1 File. All specimens are available for investigation under informal permission from IAET SB RAS. No additional permits were required for the described study. No permits were required for the described study, which complied with all relevant regulations.

Due to favorable climatic conditions of the Minusinsk basin, all the skeletal materials included in the study are characterized by a very high degree of macroscopic preservation. Fragments of long bones with a large layer of compact bone were included in the analysis.

All the ancient DNA experiments were carried out in the Laboratory of molecular paleogenetics of the Institute of Cytology and Genetics SB RAS (Novosibirsk, Russia).

### DNA extraction

DNA was extracted as previously described [11, 12]. In brief, surfaces of bones were removed mechanically, and then treated with a 7% bleach solution and irradiated by UV light (for at least 1 hour on each side of each sample). Powder was drilled from the internal compact tissue of bones. DNA was extracted from bone powder by means of incubation in a 5M guanidinium thiocyanate (GuSCN) buffer (pH 8.0) for 48 h at 65°C, followed by phenol/chloroform extraction and precipitation with isopropanol. 2–4 extractions were performed for each individual under study.

### Mitochondrial DNA analysis

The mtDNA analysis consisted of two steps. In the first step, mtDNA hyper-variable region (HVR) I was analyzed using two PCR strategies: 1) four overlapping fragments of mtDNA HVRI were amplified [13] and 2) a single long fragment was amplified by nested PCR [14] (S2 File). The HVRI fragments were sequenced directly using an ABI Prism BigDye Terminator Cycle Sequencing Ready Reaction Kit (Applied Biosystems, Foster City, CA, USA) (versions 1.1. and 3.1 for short and long fragment, respectively). Sequence data were analyzed using an ABI Prism 3130XL Genetic Analyzer (Applied Biosystems, USA) at the SB RAS Genomics Core Facility (Novosibirsk, Russia, www.sequest.niboch.nsc.ru).

Sequences were aligned with the revised Cambridge Reference Sequence of human mtDNA (rCRS) [15] by using BioEdit software version 7.0.5. Preliminary phylogenetic interpretation of sequences (based on HVRI sequences) was performed according to the current classification of mtDNA variability (mtDNA tree Build 17, http://www.phylotree.org) [16] by using the software tool HaploGrep (http://haplogrep.uibk.ac.at/) [17].

After the preliminary phylogenetic analysis of the HVRI data, informative positions in the mtDNA coding regions were analyzed, and haplogroups and sub-haplogroups were identified. Primer pairs from [18] were used to amplify mtDNA coding regions (S2 File). These coding regions were analyzed following the same methods used for HVRI. This combined two-step approach was used to clarify the phylogenetic relationships among samples and to verify the HVRI data.

Phylogenetic networks among the Tagar mtDNA haplotypes were constructed by using the Network program v.4.5.0.0. (www.fluxus-enginering.com). Phylogeographic analysis was conducted by using a database of mtDNA variability in modern Eurasian populations (S1 Table) collected from published sources.

Interpopulation differences of the Tagar population with other ancient and modern populations of Eurasia were estimated by *F*_ST_ distances [19] using the program Arlequin v.3.5.1.2 [20]. The significtableance level of Fst distances was assessed using the Monte-Carlo method, the number of interchanges was 100, and the level of significance of P = 0.05. The data of mtDNA haplogroups (and subgroups) frequencies (for interpopulation differences of the Tagar and modern Eurasian populations; S2 Table) and mtDNA HVRI sequences (for interpopulation differences of the Tagar and other ancient populations; S3 and S4 Tables) were used for this purpose. Multidimensional scaling, based on a matrix of pairwise *F*_ST_ differences, was carried out using the XLStat program (www.addinsoft.com).

Haplotype sharing analysis between Tagar population and other ancient groups was carry out as in the article [21].

### Sex determination and autosomal STR analysis

Nine autosomal STRs and the sex-determining marker amelogenin were simultaneously co-amplified by using the AmpFlSTR Profiler Plus Kit (Applied Biosystems, USA) according to the manufacturer`s instructions. Results (see S7 File) were analyzed on an ABI Prism 3130XL Genetic Analyzer (Applied Biosystems, USA) at the SB RAS Genomics Core Facility (Novosibirsk, Russia, www.sequest.niboch.nsc.ru).

### Precautions against contamination

Experimental work with the ancient material was carried out in specially equipped, isolated clean rooms using special clothes. All work surfaces and instruments were routinely cleaned with a 5% solution of bleach and irradiated by UV light. Blank controls were run in parallel with samples in all extraction and amplification procedures, to identify possible contamination. PCR with positive amplification in blanc control were discarded. MtDNA HVRI sequences were determined for all staff working in ancient DNA facility.

## Results

**Fig 2:**
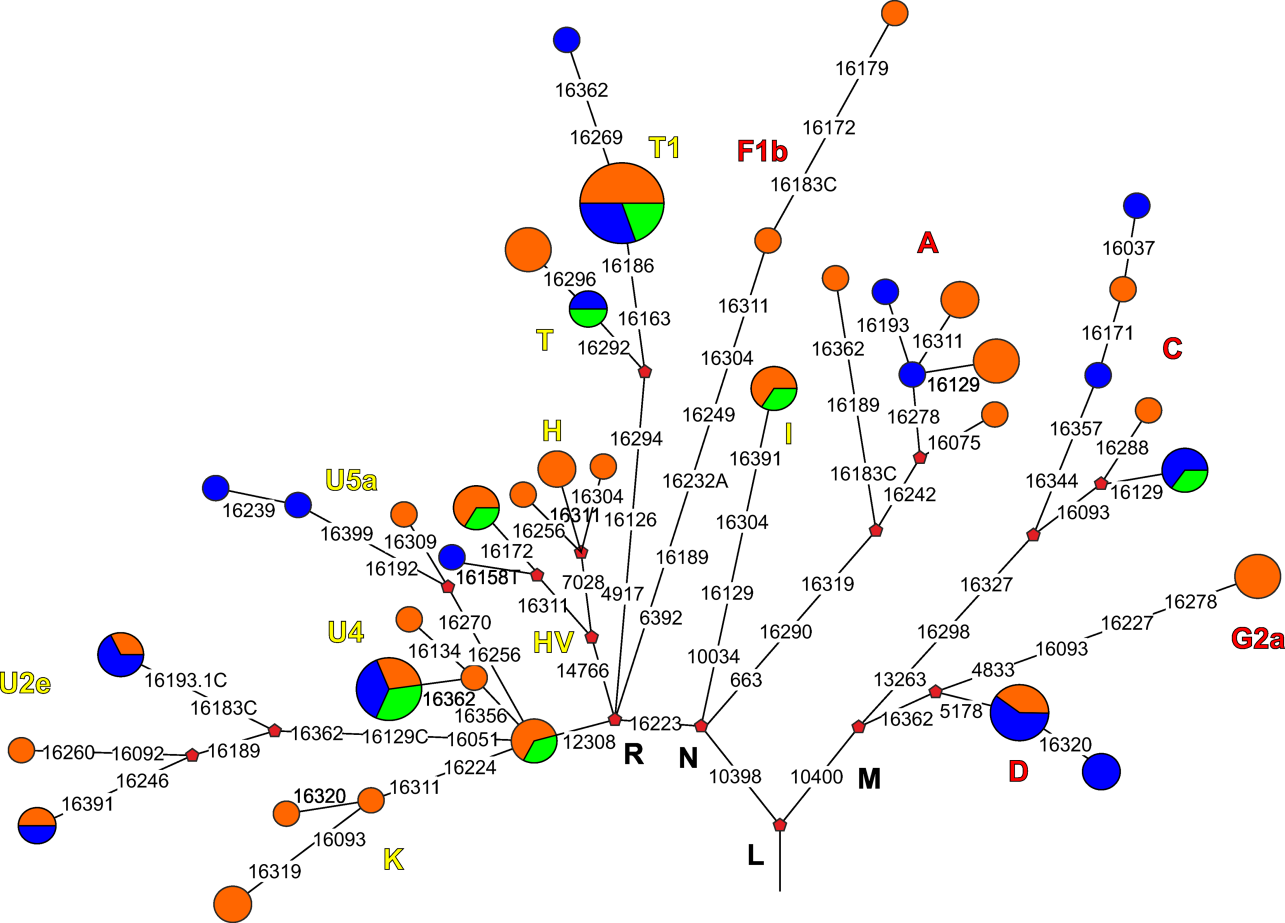
Phylogenetic tree of mtDNA lineages from the Tagar population. Color coding of the Tagar stages: *orange*—the Early Tagar stage; *blue*—the Middle Tagar Stage; *green*—the Late Tagar stage. Color of haplogroup labels: *yellow*—for Western Eurasian haplogroups; *red*—for Eastern Eurasian haplogroups.

Sequences of the mtDNA HVRI fragment (15997–16409) were obtained for 79 out of 95 ancient individuals included in the original sample set (Table 1; GenBank accession numbers MH733271-MH733349). None of the sequences obtained in ancient samples matched matched any of the researchers mtDNAs. For these individuals, data for phylogenetically informative positions in the coding region of mtDNA were also obtained. HVRI sequence data and coding-region SNPs were phylogenetically consistent for all mtDNA samples. Taken together, the HVRI sequence data and haplogroup-specific SNPs in the coding part of the mtDNA allowed us to unambiguously determine the phylogenetic position of all mtDNA samples in the study (Table 1) and build a well-supported phylogenetic tree (Fig 2). We detected 39 different HVRI haplotypes among the 79 Tagar mtDNA samples. The lineages belonged to typical Western Eurasian (H, HV6, HV*, I, K, T, U2e, U4, U5a, and U*) and Eastern Eurasian (A (including A8), C (including C4 and C5), D, G (G2a), and F (F1b)) mtDNA haplogroups. Thus, the Tagar population had a mixed mtDNA pool with substantial West Eurasian and East Eurasian components. West Eurasian lineages predominated in the overall Tagar sample both in terms of haplotype number (23 of 39 haplotypes were West Eurasian) and overall frequency in the Tagar series (51 individuals (64.6%) had West Eurasian mtDNA haplotypes, 28 (35.4%) had East Eurasian haplotypes). The West Eurasian components belonging to the T (4 lineages, 16 samples), U2e (3 lineages, 6 samples), and U4 (3 lineages, 8 samples) haplogroups were the most frequent, followed by the H (3 lineages, 4 samples), U5a (3 lineages, 3 samples), and K haplogroups (3 lineages, 5 samples). Other West Eurasian haplogroups were represented by a single haplotype (HV6, HV*, I, or U*). The East Eurasian lineages belonging to the A (6 lineages, 9 samples), C (5 lineages, 7 samples), and D (2 lineages, 7 samples) haplogroups were most frequent, followed by the F1b (2 lineages, 2 samples) and G2a (1 lineage, 3 samples) haplogroups. More than half (22 of 39) of the haplotypes were represented by a single individual in the Tagar series under study. The most frequent haplotypes were the root haplotypes of the T1 (10 individuals) and D (5 individuals) haplogroups, and haplotype 16356C-16362C belonged to the U4 haplogroup (6 individuals).

**Table 1:** Structure of mitochondrial DNA samples of Tagar individuals analyzed in this study.

Haplotype number	Samples (Tagar culture stage*)	HVR I haplotype (15997–16409)**	Status of positions in coding part of mtDNA	Haplogroup (subhaplogroup)
1	Tg38(P)	16183C-16189C-16223T-16290T-16319A-16362C	663G	A
2	Tg77(P)	16075C-16223T-16242T-16290T-16319A	663G	A8
3	Tg122(S)	16223T-16242T-16278T-16290T-16319A	663G	A8
4	Tg21(Bidj) Tg106(P) Tg120(P)	16129A-16223T-16242T-16278T-16290T-16319A	663G	A8
5	Tg102(S)	16193T-16223T-16242T-16278T-16290T-16319A	663G	A8
6	Tg1(P) Tg60(P)	16223T-16242T-16278T-16290T-16311C-16319A	663G	A8
7	Tg37(S) Tg85(T) Tg110(S)	16093C-16129A-16223T-16298C-16327T	10398G, 10400T, 13263G	C
8	Tg3(S)	16223T-16298C-16327T-16344T-16357C	10398G, 10400T, 13263G	C (C4a2a)
9	Tg2(S)	16037G-16171G-16223T-16298C-16327T-16344T-16357C	10398G, 10400T, 13263G	C (C4a2a1)
10	Tg46(P)	16171G-16223T-16298C-16327T-16344T-16357C	10398G, 10400T, 13263G	C (C4a2a1)
11	TG76(P)	16093C-16223T-16288C-16298C-16327T	10398G, 10400T, 13263G	C(C5)
12	Tg24(Bain) Tg34(S) Tg91(S) Tg100(S) Tg123(P)	16223T-16362C	10398G, 10400T, 5178A	D
13	Tg112(S) Tg121(S)	16223T-16320T-16362C	10398G, 10400T, 5178A	D
14	Tg29(P)	16189C-16232A-16249C-16304C-16311C	6392C	F1b
15	Tg33(P)	16172C-16179T-16183C-16189C-16232A-16249C-16304C-16311C	6392C	F1b (F1b1b)
16	Tg42(P) Tg56(P) Tg71(P)	16093C-16223T-16227G-16278T-16362C	10398G, 10400T, 4833G	G2a
17	Tg61(P)	16256T	14766C, 7028C	H
18	Tg108(P)	16304C	14766C, 7028C	H
19	Tg18(P) Tg23(P)	16311C	14766C, 7028C	H
20	Tg10(T) Tg69(P) Tg84(T)	16172C-16311C	14766C, 7028T	HV6
21	Tg111(S)	16158T-16311C	14766C, 7028T	HV*
22	Tg74(P) Tg80(P) Tg89(T)	16129A-16223T-16304C-16391A	10034C	I
23	Tg12(P)	16224C-16311C	12308G	K
24	Tg59(P) Tg66(Bidj)	16093C-16224C-16311C-16319A	12308G	K(K1b1a)
25	Tg54(P)	16224C-16311C-16320T	12308G	K(K1c2)
26	Tg11(P) Tg19(Bidj) Tg62(P) Tg86(T) Tg88(T) Tg94(P) Tg96(S) Tg109(S) Tg115(S) Tg116(P)	16126C-16163G-16186T-16189C-16294T	4917G	T1
27	Tg101(S)	16126C-16163G-16186T-16189C-16269G-16294T-16362C	4917G	T1
28	Tg14(P) Tg26(P) Tg49(P)	16126C-16189C-16292T-16294T-16296T	4917G	T (T2f7)
29	Tg87(T) Tg113(S)	16126C-16189C-16292T-16294T	4917G	T
30	Tg39(P) Tg92(S) Tg93(S)	16051G-16129C-16183C-(16193insC)-16362C	12308G	U2e
31	Tg70(P)	16051G-16092C-16129C-16189C-16260T-16362C	12308G	U2e
32	Tg58(P) Tg75(S)	16051G-16129C-16189C-16246G-16362C-16391A	12308G	U2e
33	Tg97(P)	16356C	12308G	U4
34	Tg57(P)	16134T-16356C	12308G	U4
35	Tg6(T) Tg9(T) Tg17(P) Tg72(S) Tg83(P) Tg117(S)	16356C-16362C	12308G	U4(U4a3)
36	Tg67(S)	16192T-16256T-16270T-16399G	12308G	U5a1
37	Tg30(Bidj)	16192T-16239T-16256T-16270T-16399G	12308G	U5a1 (U5a1h)
38	Tg99(P)	16256T-16270T-16309G	12308G	U5a
39	Tg41(P) Tg73(P) Tg119(S)	rCRS	14766T, 7028T, 12308G	U*

*Stage of Tagar culture: Early Tagar: (Bain)—Bainovo group (pre-Podgornovo), (P)—Podgornovo group, (Bidj)—Bidjinski group (post-Podgornovo); Middle Tagar: (S)—Saragash group; Late Tagar: (T)—Tes`group.

**HVRI haplotypes are given are given compared to rCRS [15].

We compared our Tagar series with two previously published small Tagar series from other burial grounds (N = 10 [10], N = 6 [4]) in terms of haplogroup composition and haplotype sharing (S3 File; S7 Table). We detected all of the haplogroups and most of the mtDNA haplotypes from series [4, 10] in our data set (9 of 12 haplotypes, represented by 13 of 16 individuals). However, 29 of 39 haplotypes were detected in the Tagar population for the first time in our work. Moreover, the A8, C4, D, HV6, K, T1, and U4 haplogroups, covering almost 60% of our Tagar series, have not been reported previously [4, 10] (S3 File; S7 Table).

**Fig 3:**
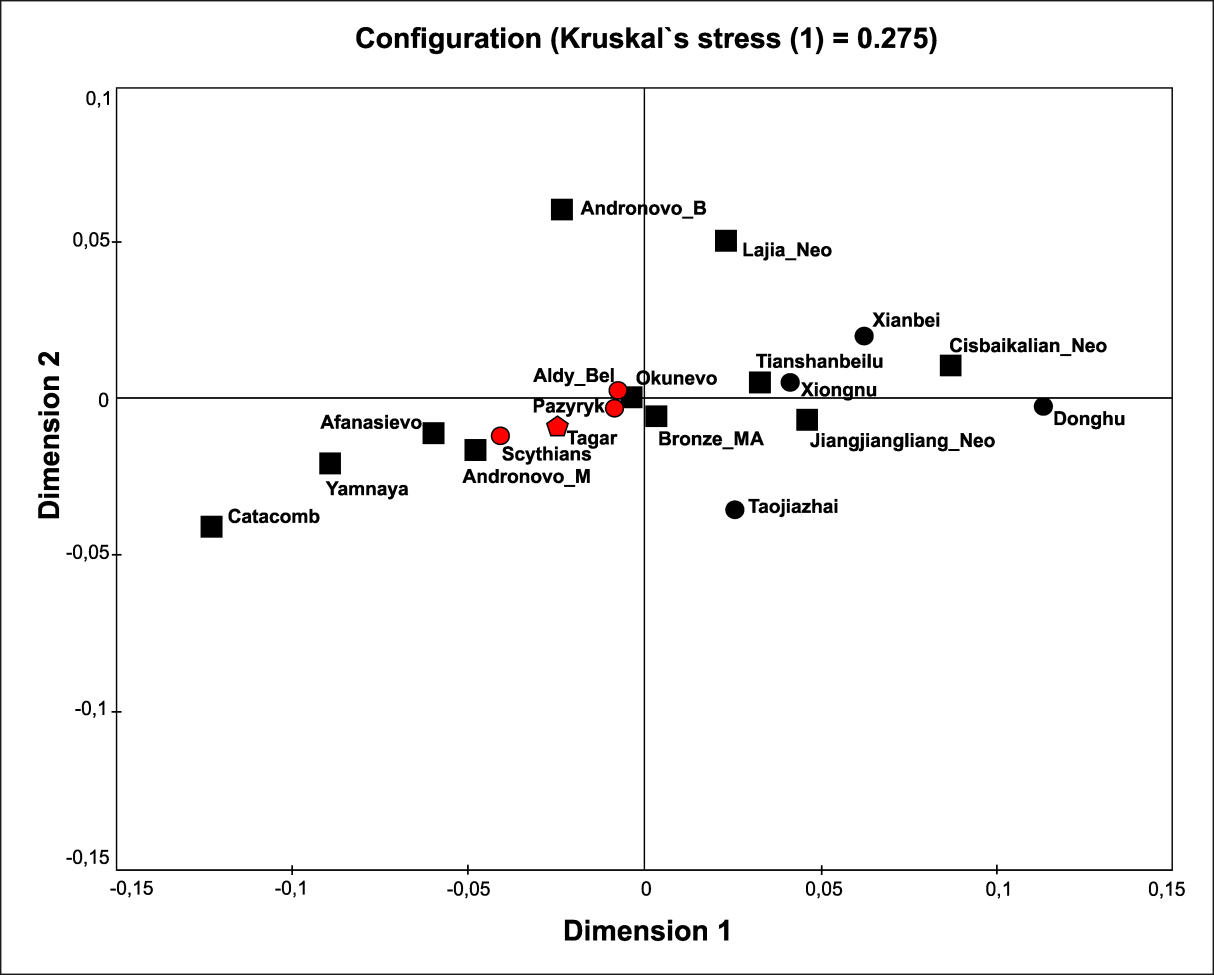
Results of multidimensional scaling based on matrix of Slatkin population differentiation (*F*_ST_) based on mtDNA HVRI sequences in the Tagar series and other ancient populations from different regions of Eurasia (details in S3 Table). Populations: **Tagar**—Tagar series (red pentagon) (this study); **Iron Age populations related with the ‘Scythian world”** (red circles): **Pazyryk**—Pazyryk culture from Altay Mountains (Russia, Kazakhstan, Mongolia) [1, 2, 4, 22–25]; **Aldy_Bel**—series from Aldy Bel culture, Arjan-2 burial complex, Tuva, Russia [4]; **Scythians**—Classic Scythians from North Pontic region [3, 4, 26]; **Neolithic and Bronze Age populations** (black squares): **Yamnaya**—Yamnaya culture population (Early Bronze Age) [18, 27–29]; **Catacomb**—Catacomb culture population (Bronze Age) [18, 29]; **Afanasievo**—Afanasievo culture population from the Minusinsk Basin (Early Bronze Age) [27, 30]; **Okunevo**—Okunevo culture population from the Minusinsk basin (Bronze Age, pre-Andronovo time); **Andronovo_B**—Andronovo time population from West-Siberian forest-steppe zone [31]; **Andronovo_M**—Andronovo culture population from Minusinsk basin [10]; **Cisbaikalian_Neo**—Serovo and Glazkovo cultures from Cis-Baikal region, Russia (Neolithic and Bronze Age) [32]; **Tianshanbeilu**—Tianshanbeilu site, eastern Xinjiang, China, Bronze Age (1900–1300 YBC) [33]; **Bronze_MA**—Middle Bronze Age population from the Mongolian Altai [34]; **Lajia_Neo**—population from the Lajia site, Qinghai, northwestern China (3800–3400 YBP) [35]; **Jiangjialiang_Neo**—Neolithic population from the Jiangjialiang site, North China [36]; **Iron Age populations not related with the ‘Scythian world”** (black triangles): **Xiongnu**—Xiongnu population from Mongolia and Transbaikalia [12, 37, 38]; **Taojiazhai**—Taojiazhai site, Qinghai, northwestern China (1900–1700 YBP) [39]; **Dondhu**—Donghu population from Jinggouzi site, Inner Mongolia, northern China (~2500 YBP) [40].

**Fig 4:**
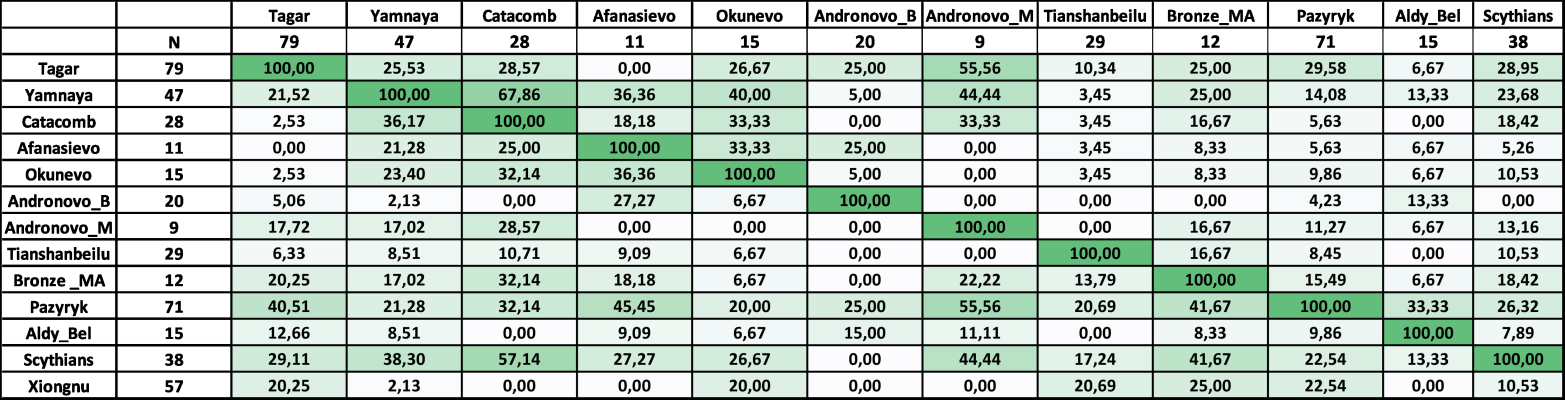
Results of haplotype sharing analysis. Percentage values of the relative shared haplotypes between the Tagar and other ancient populations. Detailed data in the S5 Table. For ascription of the populations, see Fig 3.

We compared mtDNA diversity (using a multidimensional scaling analysis of *F*_ST_ values, haplotype sharing analysis, and phylogeographic analysis) between the Tagar group and several ancient populations of Eurasia, including both earlier Neolithic and Bronze Age populations and the approximately synchronous Iron Age groups (using mtDNA HVRI sequences). Accounting for the substantial Western and Eastern Eurasian components in the Tagar gene pool, we used ancient groups from the western and eastern parts of the Eurasian steppe belt in addition to populations from Southern Siberia and adjacent regions for comparison (Figs 3 and 4; S3 and S5 Tables).

Among the Neolithic and Bronze Age groups, we detected relatively close similarity between our Iron Age Tagar population and Bronze Age Okunevo and Andronovo populations from the same territory (Minusinsk basin), followed by Afanasievo population from the Minusimsk Basin and Middle Bronze Age population from the Mongolian Altai Mountains (the region adjacent to the Minusinsk basin) (Figs 3 and 4; S3 and S5 Tables) (but see Discussion concerning the small sample size for the Minusinsk Bronze Age series). Other chronologically earlier groups from both the western and eastern parts of the Eurasian steppe belt differed substantially from the Tagar populations in terms of mtDNA variation. In the MDS plot (Fig 3), the Tagar population was positioned between chronologically earlier predominantly West Eurasian (Yamnaya and Catacomb groups) and East Eurasian (East Siberian populations and other Asian groups) populations.

Among the Iron Age populations (i.e., approximately synchronous with Tagar), the Tagar group showed a relatively close mtDNA sequence similarity with groups representing the “Scythian World” (Eurasian steppe nomadic groups of the Scythian period with specific “Scythian-like” elements in the material culture) (Figs 3 and 4; S3 and S5 Tables). We observed the greatest similarity (low *F*_ST_ and close positions on the MDS plot) between our overall Tagar series and Scythians from the North Pontic region [3, 4, 26], followed by Scythian-period groups, including the South Siberia–Pazyryk population from the Altai Mountains and the Aldy-Bel population from Tuva (Russian Federation) [4]. Iron Age nomadic groups from the eastern part of the Eurasian steppe belt, such as Xiongnu [12, 37, 38] and Donghu [40], are highly differentiated with respect to the mtDNA pool structure from the Tagar and from other groups of the “Scythian World.”

**Fig 5:**
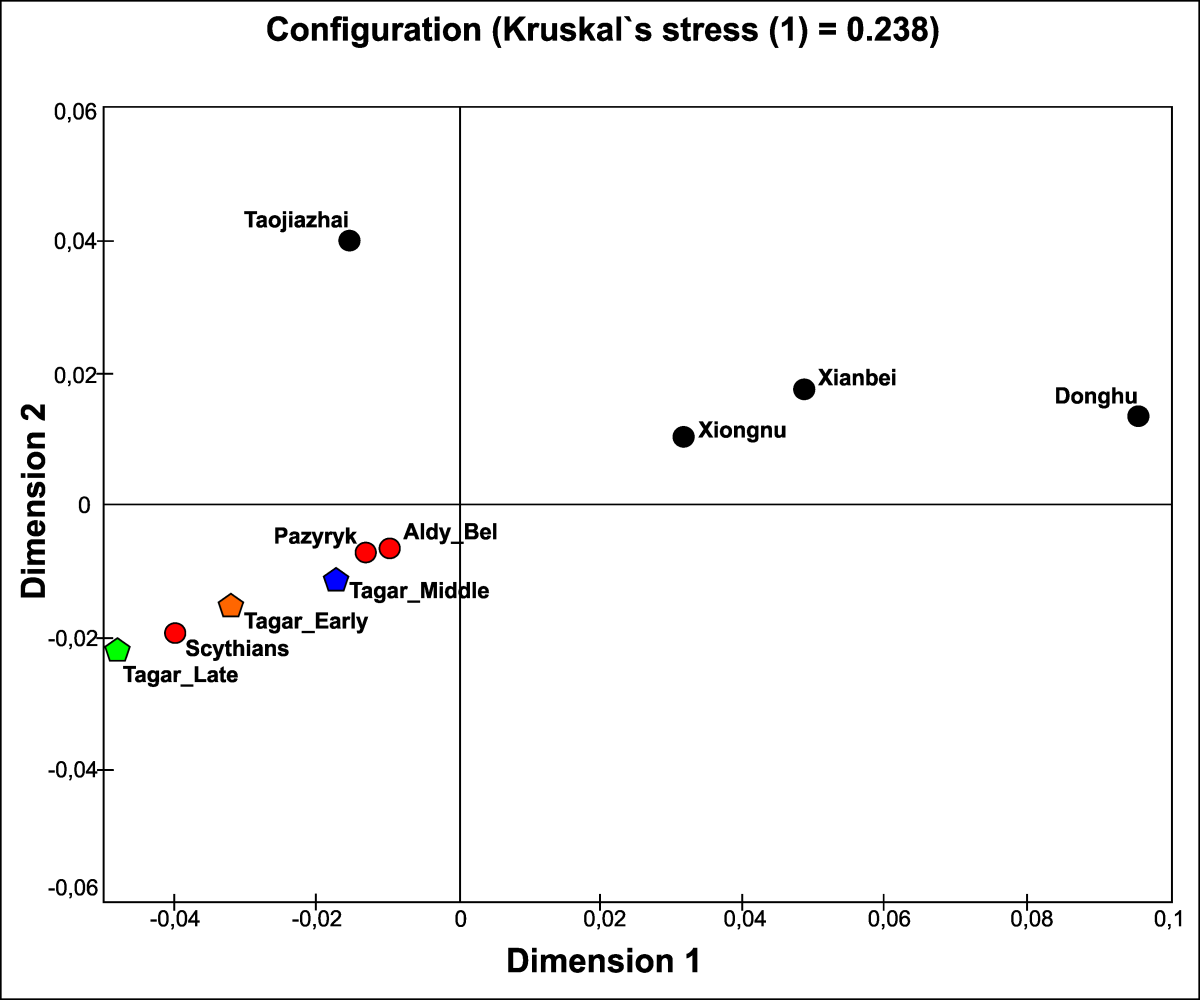
Results of multidimensional scaling based on matrix of Slatkin population differentiation (*F*_ST_) based on mtDNA HVRI sequences in the series from three main stages in the progression of the Tagar culture and other Iron Age populations from different regions of Eurasia (details in S4 Table). Populations: **Tagar_Early**—population of early stage of the Tagar culture (mainly the Podgornovo type) (orange pentagon) [this study]; **Tagar_Middle**—population of middle stage of the Tagar culture (Saragash type) (blue pentagon) (this study); **Tagar_Late**—population of late stage of the Tagar culture (Tes`type) (green pentagon) (this study); Xianbei—Xianbei population [41, 42]. For ascription of other populations, see Fig 3.

**Fig 6:**
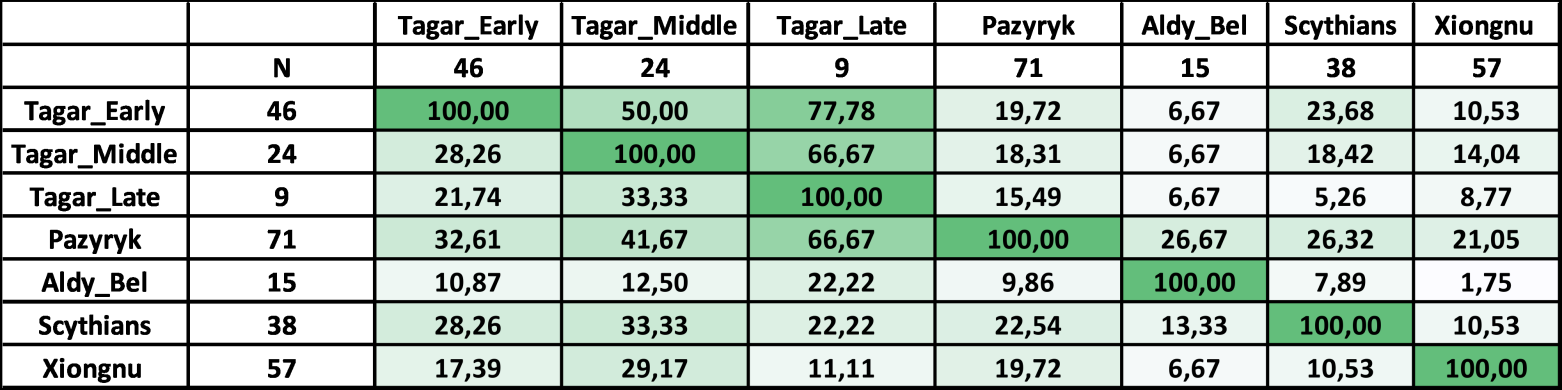
Results of haplotype sharing analysis. Percentage values of the relative shared haplotypes between the series from three main stages in the progression of the Tagar culture and other Iron Age populations of Eurasia. Detailed data in the S6 Table. For ascription of the populations, see Fig 3.

Our series (N = 79) included individuals representing all three main stages in the progression of the Tagar culture (although the sample sizes for the Early and Middle Tagar culture periods were larger: Early stage (N = 46), Middle stage (N = 24), and Late stage (N = 9)); accordingly, we compared the mtDNA sequences of these chronologically successive Tagar groups with each other and with the sequences of other Iron Age nomadic populations to infer the dynamics of the mtDNA pool structure of Tagar populations and to detect potential external influences on their genetic composition at different cultural stages (Figs 5 and 6; S4 and S6 Tables).

The positions of non-Tagar Iron Age groups in the MDS plot were correlated with their geographic position within the Eurasian steppe belt and with frequencies of Western and Eastern Eurasian mtDNA lineages in their gene pools. Series from chronological Tagar stages (similar to the overall Tagar series) were located within the genetic variability (in terms of mtDNA) of Scythian World nomadic groups (Figs 5 and 6; S4 and S6 Tables). Specifically, the Early Tagar series was more similar to western nomads (North Pontic Scythians), while the Middle Tagar was more similar to the Southern Siberian populations of the Scythian period. The Late Tagar group (Tes`culture) belonging to the Early Xiongnu period had the “western-most” location on the MDS plot with the maximal genetic difference from Xiongnu and other eastern nomadic groups (but see Discussion concerning the low sample size for the Tes`series).

**Fig 7:**
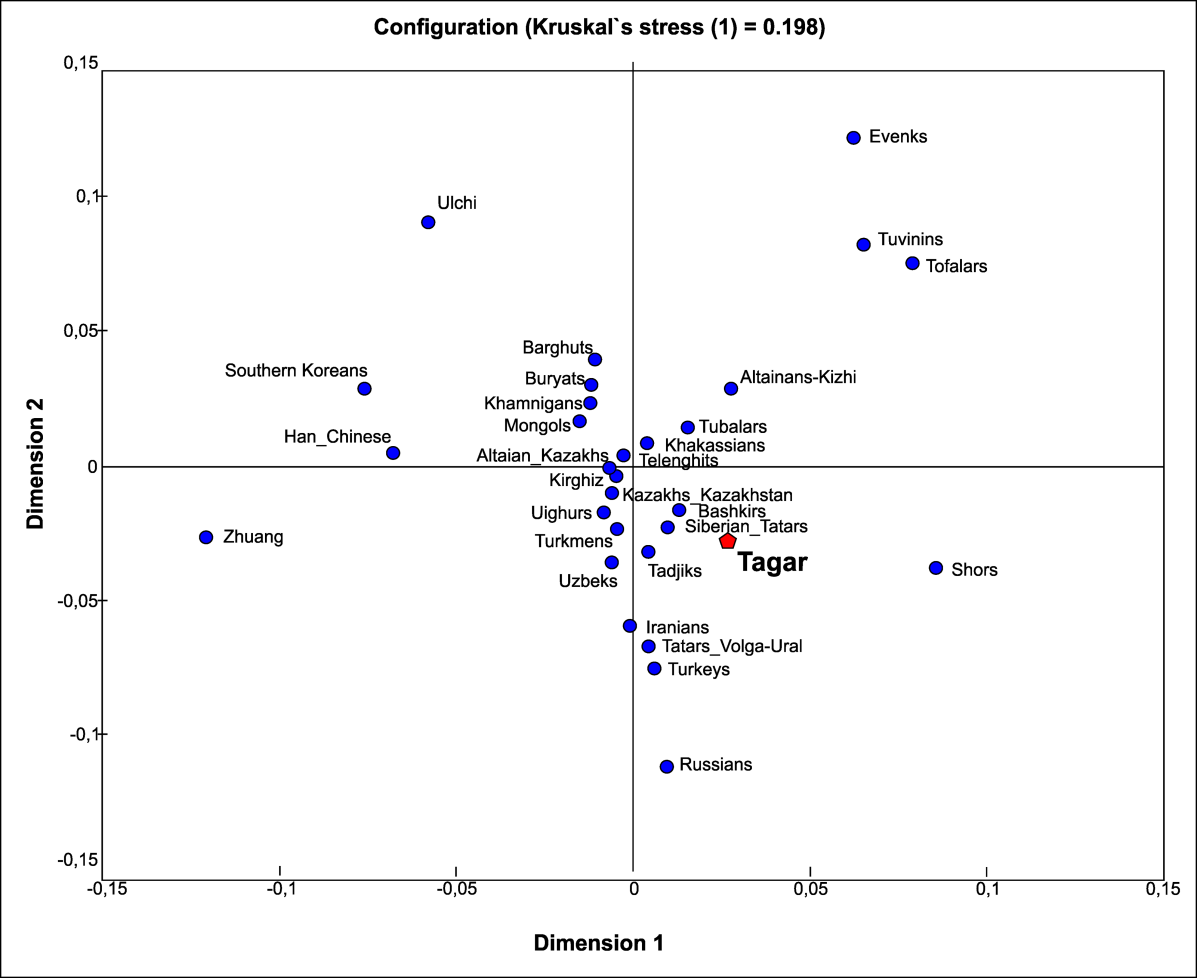
Results of multidimensional scaling based on matrix of Slatkin population differentiation (*F*_ST_) according to frequencies of mtDNA haplogroup in Tagar populations and modern populations of Eurasia (S2 Table). Populations: **Tagar** (red pentagon) (this study); Mongolian-speaking populations: **Khamnigans** (Buryat Republic, Russia) [43]; **Barghuts** (Inner Mongolia, China) [44]; **Buryats** (Buryat Republic, Southern Siberia, Russia) [43]; **Mongols** (Mongolia) [45]. Turkic-speaking populations: Tuvinians (Tuva Republic, Russia) [43]; Tofalars (Irkutsk region, Russia) [46]; Altai-Kizhi ((Altai Republic, Russia) [43, 47]; Telenghits (Altai Republic, Russia) [43,47]; Tubalars (Altai Republic) [48]; Shors (Kemerovo region, Russia) [43, 47]; Khakassians (Khakassian Rupublic, Russia) [43, 46]; Altaian Kazakhs (Altai Republic) [49]; Kazakhs (Kazakhstan, Uzbekistan) [50, 51]; Kirghiz (Kyrgyzstan) [50, 51]; Uighurs (Kazakhstan and Xinjiang) [50, 52]; Siberian Tatars (Tyumen and Omsk regions, Russia) [53]; Tatars (Volga-Ural rigion, Russia) [54]; Bashkirs (Volga-Ural rigion, Russia) [55]; Uzbeks (Uzbekistan) [51, 56]; Turkmens (Turkmenistan) [51, 56]; Nogays [57]; Turkeys [58]; other populations: Evenks [43, 46]; Ulchi [59]; Koreans (South Korea) [43]; Han Chinese [60]; Zhuang (Guangxi, China) [61]; Tadjiks (Tadjikistan) [43, 51]; Iranians [60]; Russians [62].

In a comparison of our Tagar series with modern populations in Eurasia, we detected similarity between the Tagar group and some modern Turkic-speaking populations (with the exception of the Indo-Iranian Tajik population) (Fig 7; S2 Table). Among the modern Turkic-speaking groups, populations from the western part of the Eurasian steppe belt, such as Bashkirs from the Volga-Ural region and Siberian Tatars from the West Siberian forest-steppe zone, were more similar to the Tagar group than modern Turkic-speaking populations of the Altay-Sayan mountain system (including the Khakassians from the Minusinsk basin) (Fig 7).

A phylogeographic analysis revealed both geographically widespread and specifically distributed (i.e., phylogeographically informative) mtDNA lineages in our Tagar series. We observed informative lineages among both the East Eurasian and West Eurasian parts of our Tagar series. Several lineages from the Tagar series belonged to East Eurasian mtDNA clusters, including A8, C4a2, F1b1b, and G2a, which are characteristic of modern populations in Southern Siberia and adjacent regions of Eastern Central Asia and Middle Asia.

Several lineages and subclusters originated from western areas of Eurasia and were dominant in modern and ancient populations of various parts of Europe, Caucasus, and the Near East (K, HV6, H, and other West Eurasian mtDNA haplogroups). Most of these West Eurasian clusters were present in Southern Siberia since the Bronze Age [10]. Most of these subclusters were present at low frequencies in the modern indigenous populations of Central Asia [43].

Thus, our analysis revealed the presence of distinct phylogeographical components in the Tagar mtDNA series and mixed origins of the Tagar mtDNA pool. However, several West Eurasian (lineages of haplogroups U2e, U4, and T1) and East Eurasian (lineages of haplogroups C and D) lineages from the Tagar series showed wide distributions in the territory of Western and Central Eurasia (for West Eurasian clusters) or Eastern and Central Eurasia (for East Eurasian clusters) and therefore were phylogenetically uninformative.

## Discussion

Early nomads originated in the Eurasian steppes in the beginning of the 1^st^ millennium BC and played a key role in the formation of the cultural and genetic landscape of populations of a large part of Eurasia from Eastern Europe to Eastern Central Asia during the so-called pre-Scythian, Scythian, and Xiongnu-Sarmatian times and in subsequent periods. A large number of archaeological cultures associated with early nomads have been discovered in both the west and east regions of the Eurasian steppe belt. Analyses of these archaeological materials by various methods make it possible to objectively reconstruct the important ethnogenetic processes involving early nomads. Paleogenetic study of the anthropological remains of early nomads is one of the most promising areas of research at the moment. Analyses of the genetic composition of both eastern [1, 2, 12, 37, 38] and western [3, 26] groups of early nomads of the Scythian and Xiongnu-Sarmatian epoch as well as the comparative analysis of early nomads from different regions [4] have been published. However, only a small series of individuals have been investigated for most early nomadic populations, even at the level of mitochondrial DNA, with the exception of the Pazyryk culture from the Altai Mountains (Scythian time) and Xiongnu, for which fairly representative mtDNA samples have been published.

### Representativity of the obtained results

We analyzed a number of mtDNA samples (N = 79) from representatives of the Tagar culture from the Minusinsk basin (the middle course of the Yenisei River, Sayan Mountains, Southern Siberia). The Tagar culture existed in the Minusinsk basin for a long time, at least from the 8^th^ to 1^st^ century BC, i.e., during the pre-Scythian, Scythian, and Early Xiongnu-Sarmatian periods. Only a small number of samples from the Tagar culture (belonging to one chronological period [4] or without indication of the Tagar cultural stage [10]) have been published previously. Our series includes samples from all the main chronological stages of the Tagar culture: Early Tagar, Middle Tagar, and Late Tagar periods (see S1 and S4 Files). These new results place the Tagar culture within the most well-studied (in terms of mtDNA diversity) early nomadic groups from Siberia.

Our work is a good example of the correlation between the number of mtDNA samples and the quality, generalizability and representativity of mtDNA diversity data for an ancient population. More than 74% of HVRI haplotypes and several haplogroups (covering almost 60% of individuals analyzed in this study) found in our Tagar series were absent from previously published data (N = 16) [4, 10].

For a mtDNA series including N samples, under a simple binomial approximation, we cannot rule out the possibility that some undetected mtDNA clusters existed in the population at a frequency *p* with (1–*p*)^N^ = 0.05. For N = 16 (data from [4, 10]), *p* = 0.171. Thus, previously published Tagar mtDNA data could occasionally not include clusters that were among the most highly represented in the gene pool of this ancient population. In particular, we observed haplogroups T1, U4, and A8, which were absent from previously published data, in our more representative series at frequencies of 13.9%, 10.1%, and 10.1%, respectively. For our series (N = 79), *p* = 0.037. With a high probability, we identified all of the main clusters of mtDNA present in the gene pool of the Tagar culture population in the Minusinsk basin. Thus, we obtained substantially more representative mtDNA diversity data for the Tagar population than were previously available.

### Mitochondrial DNA diversity and genetic relationships of the Tagar population

According to our results, the Tagar populations show a similar mtDNA pool structure to those of other Iron Age populations representing the “Scythian World” (Figs 3 and 4; S3 and S5 Tables). These results are consistent with those of previous results [4] (note that the Tagar group was under-represented in this previous study). When analyzing the whole Tagar series, the greatest similarity was detected between the Tagar and Classic Scythians from the North Pontic region (Figs 3 and 4; S3 and S5 Tables). Interestingly, the close similarity in mtDNA pool structure is in agreement with the results of a craniometric study of these populations; both female and male series from the Tagar population show high craniofacial similarity with several local groups of Classic Scythians from the North Pontic region [63, 64].

It has been suggested that the genetic similarity between the western and eastern early nomadic Scythian-like groups reflects the multiregional (local) origin of these groups with similar genetic components [4]. According to this scenario, West Eurasian components of the mtDNA pool, which are largely responsible for the similarity in genetic composition (with respect to mtDNA) between eastern and western early nomads, were most likely introduced into Southern Siberia by migratory flows from the western part of Eurasia that occurred during the Bronze Age and are well documented based on archaeological data. There were two main suggested migration waves to the Minusinsk basin during the Bronze Age: (1) the migration of populations from Eastern Europe (possibly related to the Yamnaya culture), resulting in the rise of the Afanasievo culture in the Altai and Sayan Mountains during the Early Bronze Age (3^rd^ millennium BC) [9], and (2) the migration of the Andronovo (Fedorovo) culture population from adjacent regions of the Eurasian steppe (from modern Kazakhstan territory) during the 1^st^ half of the 2^nd^ millennium BC (Middle Bronze Age) [9, 65].

Our results are not inconsistent with the assumption of a probable role of gene flow due to the migration from Western Eurasia to the Minusinsk basin in the Bronze Age in the formation of the genetic composition of the Tagar population. Particularly, we detected many mtDNA lineages/clusters with probable West Eurasian origin that were dominant in modern populations of different parts of Europe, Caucasus, and the Near East (such as K and HV6) in our Tagar series based on a phylogeographic analysis.

To assess the potential influence of Bronze Age populations in the Minusinsk basin (and the related migration wave from west of the Eurasian steppe) on the formation of the genetic composition of the Tagar population we compared our Tagar series with several chronologically preceded ancient populations: 1) Early Bronze Age (pre-Andronovo) Afanasievo and Okunevo culture populations from Minusinsk basin and Ealy Bronze Age Yamnaya culture populations from Eastern Europe (since numerous data from archaeology, physical anthropology, and genomic analyses support the hypothesis that the Yamnaya populations were the ancestors of the Afanasievo group); 2) Middle Bronze Age populations, related with Andronovo migration wave during the 1^st^ half of the 2^nd^ millennium BC—Andronovo culture population from the Minusinsk basin and Andronovo-time population from the adjacent Baraba forest-steppe region.

We detected relatively low genetic distances between our Tagar population and two Bronze Age populations from the Minusinsk basin—the Okunevo culture population (pre-Andronovo Bronze Age) and Andronovo culture population, followed by Afanasievo population from the Minusinsk Basin and Middle Bronze Age population from the Mongolian Altai Mountains (the region adjacent to the Minusinsk basin) (Figs 3 and 6; S3 and S5 Tables). Among West Eurasian part of our Tagar series we also observed haplogroups/sub-haplogroups and haplotypes shared with Early and Middle Bronze Age populations from Minusinsk Basin and western part of Eurasian steppe belt (Fig 4; S5 Table). Thus, our results suggested a potentially significant role of the genetic components, introduced by migrants from Western Eurasia during the Bronze Age, in the formation of the genetic composition of the Tagar population. It is necessary to note the relatively small size of available mtDNA samples from the Bronze Age populations of Minusinsk basin; accordingly, additional mtDNA data for these populations are required to further confirm our inference.

However, based on our mtDNA data, we cannot exclude the possibility that some West Eurasian clusters of mtDNA were introduced (or reintrodused) to Southern Siberian Iron Age populations more recently, independent of the Bronze Age migration waves discussed above. In this regard, it is interesting to note some specific West Eurasian mtDNA lineages shared between eastern and western populations of the Scythian World. For example, a lineage with rCRS HVRI haplotype belonged to haplogroup U (haplotype number 39 in Table 1; Fig 2) was identified both in the Tagar population of Minusinsk basin (five individuals from two independent studies—this work; [10]) and in Classic Scythians from the North Pontic region [3]. This lineage is absent from the published mtDNA data for Bronze Age populations, both from the territory of Southern Siberia (including the Minusinsk basin) and the central and western regions of the Eurasian steppe belt. The source of this Western Eurasian component in the Tagar population gene pool is unclear.

Another substantial part of the mtDNA pool of the Tagar and other eastern populations of the Scythian World is typical of populations in Southern Siberia and adjacent regions of Central Asia (autochthonous Central Asian mtDNA clusters). Most of these components belong to the East Eurasian cluster of mtDNA haplogroups. Moreover, the role of each of these components in the formation of the genetic composition of subsequent (to the present) populations in South Siberia and Central Asia could be very different. In this regard, cluster C4a2a (and its subcluster C4a2a1), and haplogroup A8 are of particular interest.

Lineages in the C4a2a (and C4a2a1) cluster represent a significant portion of the haplogroup C diversity in our Tagar series (three out of five lineages). Interestingly, in the Early Iron Age, the mtDNA cluster C4a2a (including C4a2a1) did not appear to be widely distributed in Southern Siberia and Central Asia: these lineages were not detected in other populations of Scythian and Xiongnu-Sarmatian times, both in the territory of the Altai-Sayan mountain system and beyond. Only a single mtDNA line belonging to the related cluster C4a2 was detected in the Mongolian Xiongnu [36]. This differs substantially from modern populations: the C4a2a lineages (including mainly C4a2a1) are now a typical component of the mtDNA pool of the modern Turkic-speaking and Mongolian-speaking populations of Central Asia (including Southern Siberia) (S5 File). Therefore, a major increase in the role of this lineage in the genetic composition of Central Asian populations occurred over the past 2000 years. Descendants of the Tagar population (or genetically related populations) could potentially play a substantial role in this process (see more details in S5 File).

**Fig 8:**
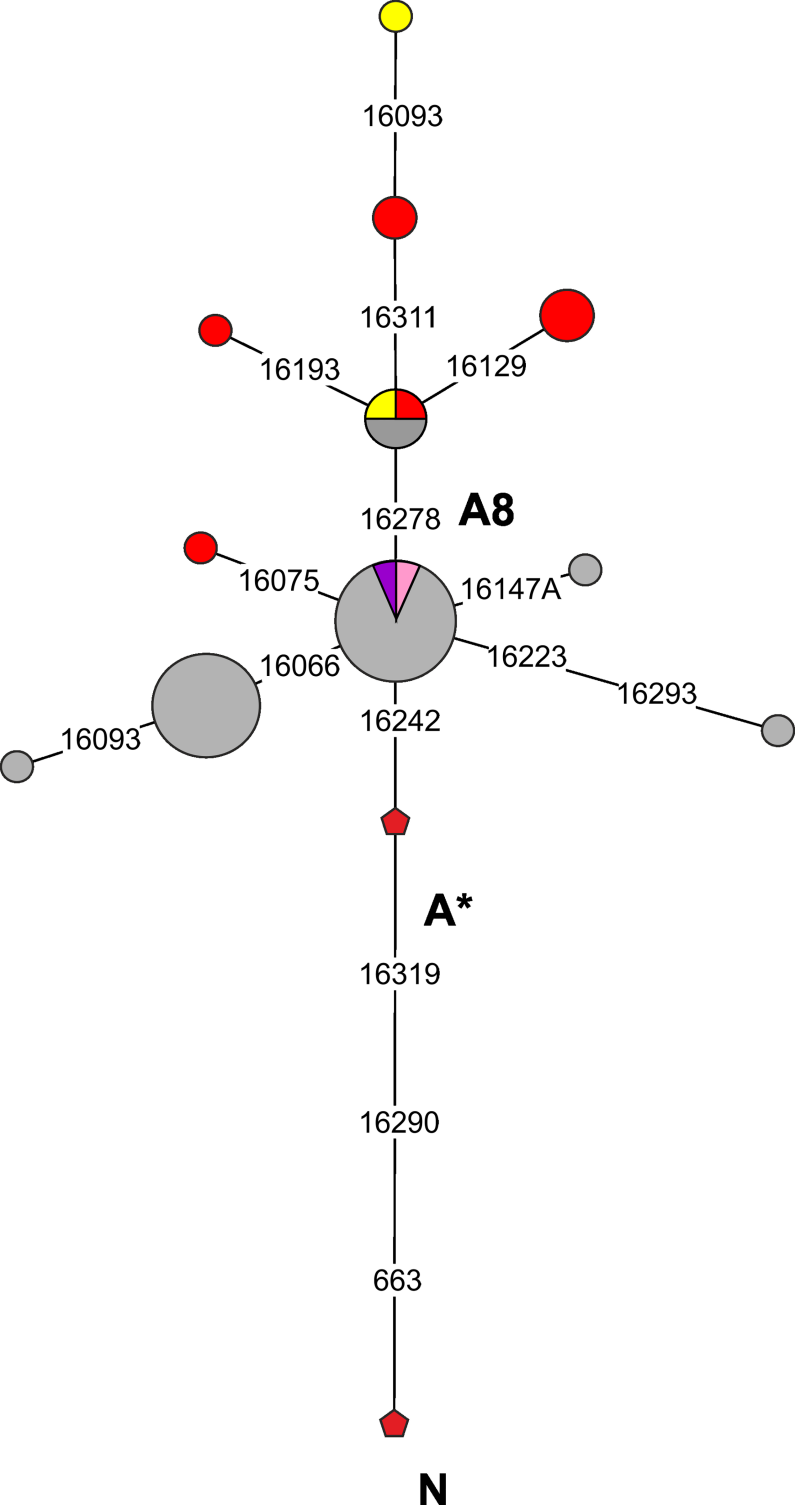
Phylogenetic tree of mtDNA haplogroup A8 lineages from modern and ancient populations of Eurasia. Ascription to the populations showed by colors: *grey*—modern populations of Eurasia [43, 44, 47, 49, 50, 56, 57, 59, 66–70] (see S6 File for details); *red*—Tagar culture (this study); *yellow*—Pazyryk culture [4]; *pink*—Aldy-Bel culture [4]; *purple*—Okunevo culture [27, 28].

Unlike C4a2a, the role of A8 in the gene pool of the population of Southern Siberia has reduced substantially over the past ~2000 years. This cluster was widely represented in Scythian-Siberian groups, including the Tagar, Pazyryk and Aldy Bel culture populations (this study and [4]) (Fig 8; S6 File). In modern Eurasian population haplogroup A8 is a rare component of the mtDNA gene pool and is distributed with low frequencies mainly in Eastern (including the south of Siberia) and Western Central Asia (and it is very rare in Western Eurasia [66]) (Fig 8; S6 File). Interestingly, most variants of A8 found in the ancient Scythian-Siberian populations are characterized by a common haplotype with a C16278T substitution and obviously belong to a subcluster of haplogroup A8 that has not been annotated to date. Conversely, A8 lineages lacking С16278T are dominant in modern populations of Central Asia (see details in S6 File).

These components, e.g., haplogroups A8 and C4a2a (C4a2a1), in the gene pool of populations in the Early Iron Age and more recent periods in the western regions of the Eurasian steppe belt are potential markers of the influence of the eastern populations during the Scythian period on western populations. According to the results of Unterlander et al. [4], East Eurasian mtDNA components in the Western Eurasian steppe belt increased during the Early Iron Age. However, their earlier penetration into the western regions of the Eurasian steppe belt cannot be excluded. For example, lineages of the C4a2 (but not the C4a2a or C4a2a1) haplogroup have been identified in the Neolithic populations of the North Pontic region in Ukraine [29].

### Genetic features of successive Tagar groups

We compared successive Tagar groups (Early, Middle, and Late Tagar) with each other and with other Iron Age nomadic populations to evaluate changes in the mtDNA pool structure. Despite the genetic similarity between the Early and Middle Tagar series and Scythian World nomadic groups (Figs 5 and 6; S4 and S6 Tables), there were some peculiarities. For example, the Early Tagar series was more similar to North Pontic Classic Scythians, while the Middle Tagar samples were more similar to the Southern Siberian populations of the Scythian period (i.e., completely synchronous populations of regions neighboring the Minusinsk basin, such as the Pazyryk population from the Altay Mountains and Aldy-Bel population from Tuva).

We observed differences in the mtDNA pool structure between the Early and the Middle chronological stages of the Tagar culture population, as evidenced by the change in the ratio of Western to Eastern Eurasian mtDNA components. The contribution of Eastern Eurasian lineages increased from about one-third (34.8%) in the Early Tagar group to almost one-half (45.8%) in the Middle Tagar group.

At the level of mtDNA haplogroups, we detected a decrease in the diversity of phylogenetic clusters during the transition from the Early Tagar to the Middle Tagar. This decline in diversity equally affected the West Eurasian and East Eurasian components of the Tagar mtDNA pool. It should be noted that this decrease can be partially explained by the smaller number of Middle Tagar than Early Tagar samples. Under a simple binomial approximation the mtDNA clusters, observed at frequencies of 6.3% and 11.7%, could be lost by chance in our Early (N = 46) and Middle (N = 24) Tagar samples, respectively. However, the simultaneous lack of several such clusters, with a total frequency in the gene pool of the Early group of 34.8%, is unlikely.

Using a haplotype sharing analyses, we obtained similar results (Fig 6; S6 Table). Exactly half of the mtDNA lineages in the Middle Tagar series are identical to Early Tagar lineages. Moreover, almost all of the unique (non-identical) mtDNA lineages from the Middle Tagar series were phylogenetically closely related to the Early Tagar lineages and belonged to the same mtDNA phylogenetic cluster (except for one, lineage 16158T-16311C, HV*). On the other hand, 23 out of 29 mtDNA haplotypes, representing 72% of the Early Tagar series, were missing from the Middle series.

Thus, our results confirm the genetic continuity between the Early and Middle Tagar groups. Additionally, we detected a reduction in mtDNA diversity in the Tagar gene pool at the level of haplogroups and haplotypes. We did not find evidence for a migration wave or a change in the direction of interpopulation genetic interactions during this period.

The observed reduction in the genetic distance between the Middle Tagar population and other Scythian-like populations of Southern Siberia(Fig 5; S4 Table), in our opinion, is primarily associated with an increase in the role of East Eurasian mtDNA lineages in the gene pool (up to nearly half of the gene pool) and a substantial increase in the joint frequency of haplogroups C and D (from 8.7% in the Early Tagar series to 37.5% in the Middle Tagar series). These features are characteristic of many ancient and modern populations of Southern Siberia and adjacent regions of Central Asia, including the Pazyryk population of the Altai Mountains. We did not obtain strong evidence for an intensification of genetic contact between the population of the Minusinsk basin and the Altai Mountains in the Middle Tagar period compared with the Early Tagar period. Although, several archaeologists have found evidence for the intensification of contact at the level of material culture, namely, a cultural influence of the population of the Altai Mountains (represented by the Pazyryk population) on the population of the Minusinsk basin (the Saragash Tagar group) [6, 71, 72].

Another important issue is the change in the genetic structure of the Tagar population during the transition from the Middle (Saragash) to the Late (Tes`) stage. The Late Tagar stage refers to the Xiongnu period. Many archaeologists suggest that the formation of the Tes`stage involved the direct cultural influence of the Xiongnu and/or related groups of nomads from more eastern regions of Central Asia [71, 73]. Some archaeologists have even suggested renaming the Tes`stage in the Tes`culture [71], emphasizing the role of new eastern cultural elements. If this influence also existed at the genetic level, then we would expect to observe new genetic elements in the Tes`gene pool, particularly those of East Eurasian origin.

In this study, we only investigated a small series of Tes`mtDNA samples. We do not have a complete understanding of the genetic composition of the Tes`population. However, our results are of interest in the context of the possible influence of eastern nomads during this period. The Late Tagar group (Tes`stage) was most distantly related to Xiongnu and other eastern nomadic groups (see MDS plot at Fig 5; S4 Table). Moreover, eight of the nine Tes`mtDNA samples belonged to the West Eurasian mtDNA cluster. Furthermore, all of the mtDNA lineages detected in our small Late Tagar series were also present in the series from earlier Tagar groups (Figs 2 and 6; Table 1; S6 Table). Thus, we do not have any evidence for an influence of a genetically divergent population from more eastern regions of Central Asia on the population of the Minusinsk basin in this period. To assess the level of genetic continuity and identify any external genetic influence, a significant increase in the Tes`series is necessary.

Based on our results, we can preliminarily conclude that there was genetic continuity, at least partially, between the Early, the Middle and the Late Tagar populations. We did not find evidence of extensive gene exchange between the Tagar population and any genetically distinct (with respect to the mtDNA pool) human groups.

## Supporting information

S1 File Description of paleoanthropological materials analyzed in this study. (DOCX)

S2 File PCR-primers used for amplification of mtDNA fragments. (DOCX)

S3 File Previously published data on mtDNA structure from Tagar specimens. (DOCX)

S4 File Characteristics of the main stages in the progression of the Tagar culture (archaeological features and chronology). (DOCX)

S5 File Frequency of mtDNA haplogroup C4a2a* and C4a2a1 in some modern populations of Siberia and adjacent regions of Eurasia. (DOCX)

S6 File Distribution of mtDNA A8 haplogroup lineages in modern and ancient human populations of Eurasia. (DOCX)

S7 File Autosomal STR-loci allelic profiles and results of sex determination of the Tagar individuals. (DOCX)

S1 Table List of modern Eurasian populations used for phylogeographic analysis. (XLSX)

S2 Table Interpopulation differences (matrix of Slatkin population differentiation *F*_ST_) between the Tagar population (overall sample) and modern Eurasian populations based on mtDNA haplogroup frequencies and MDS coordinates. (XLSX)

S3 Table Interpopulation differences (matrix of Slatkin population differentiation *F*_ST_) between the Tagar population (overall sample) and other ancient Eurasian populations based on mtDNA HVRI sequences and MDS coordinates. (XLSX)

S4 Table Interpopulation differences (matrix of Slatkin population differentiation *F*_ST_) between the in the series from three main stages in the progression of the Tagar culture and other Iron Age populations from different regions of Eurasia based on mtDNA HVRI sequences and MDS coordinates. (XLSX)

S5 Table Results of the haplotype sharing analysis (HSA) between the Tagar population (overall sample) and other ancient populations of Eurasia. (XLSX)

S6 Table Results of the haplotype sharing analysis (HSA) between the series from three main stages in the progression of the Tagar culture and other Iron Age populations of Eurasia. (XLSX)

S7 Table Comparison of mtDNA haplogroup composition between out Tagar series and previously published Tagar samples. (XLSX)
