# Supplementary material for: Maternal genetic features of the Iron Age Tagar population from Southern Siberia (1st millennium BC)
Source: PLoS One. 2018 Sep 20;13(9):e0204062. doi: 10.1371/journal.pone.0204062 (PMC6147448; doi:10.1371/journal.pone.0204062)
Supplement: S1 File — (DOCX) [file pone.0204062.s001.docx]

**S1 File. Description of paleoanthropological materials analyzed in this study.**

**Samples from Early Stages of the Tagar Culture.**

| N | Laboratory Code (Tagar Group*) | Burial ground | Description | Bone fragment | Age of Individual |
| --- | --- | --- | --- | --- | --- |
| 1 | Tg1(P) | Tepsej-8 | Kurgan 10, main burial | Tibia | Adult |
| 2 | Tg11(P) | Tepsej-8 | Kurgan 12, burial 7 | Femur | Child |
| 3 | Tg12(P) | Tepsej-8 | Kurgan 12, burial 6 | Femur | Child |
| 4 | Tg14(P) | Tepsej-9 | Kurgan 5, burial 2 | Tibia | Adult |
| 5 | Tg17(P) | Tepsej-9 | Kurgan 3, burial 2 | Tibia | Adult |
| 6 | Tg18(P) | Tepsej-9 | Kurgan 2, burial 1 | Femur | Adult |
| 7 | Tg19(Bidj) | Kichik-Kyuzyur | Kurgan 2, burial 2 | Humerus | ? |
| 8 | Tg21(Bidj) | Kichik-Kyuzyur | Kurgan 4, burial 4 | Tibia | ? |
| 9 | Tg23(P) | Tepsej-9 | Kurgan 1, burial 4, Skeleton 1 | Tibia | Adult |
| 10 | Tg24(Bain) | Sovetskaya Hakasiya | Kurgan 2, main burial | Tibia | ? |
| 11 | Tg26(P) | Tepsej-9 | Kurgan 5, burial 1, Skeleton 2 | Tibia | Adult |
| 12 | Tg29(P) | Tepsej-9 | Kurgan 3, burial 1 | Tibia | Adult |
| 13 | Tg30(Bidj) | Barsuchiha-6 | Kurgan 6, burial 1 | Femur | ? |
| 14 | Tg33(P) | Barsuchiha-6 | Kurgan 5, burial 1 | Tibia | ? |
| 15 | Tg38(P) | Novaya chernaya-1 | Kurgan 3, burial 1 Skeleton 1 | Tibia | ? |
| 16 | Tg39(P) | Novaya chernaya-1 | Kurgan 4, burial 1 | Femur | Adult |
| 17 | Tg41(P) | Novaya chernaya-1 | Kurgan 22, burial 1 | Tibia | Adult |
| 18 | Tg42(P) | Novaya chernaya-1 | Kurgan 4, burial 2 | Femur | Adult |
| 19 | Tg46(P) | Novaya chernaya-1 | Kurgan 5, burial 3 | Tibia | ? |
| 20 | Tg49(P) | Novaya chernaya-1 | Kurgan 3, burial 1 Skeleton 2 | Femur | ? |
| 21 | Tg54(P) | Podgornoe ozero | Kurgan 32, burial 3 | Tibia | ? |
| 22 | Tg56(P) | Podgornoe ozero | Kurgan 35, burial 2 | Tibia | Adult |
| 23 | Tg57(P) | Podgornoe ozero | Kurgan 33, burial 3, Skeleton 1 | Tibia | Adult |
| 24 | Tg58(P) | Podgornoe ozero | Kurgan 33, burial 3, Skeleton 2 | Tibia | ? |
| 25 | Tg59(P) | Podgornoe ozero | Kurgan 37, burial 1 | Humerus | ? |
| 26 | Tg60(P) | Podgornoe ozero | Kurgan 32, burial 2 | Tibia | ? |
| 27 | Tg61(P) | Podgornoe ozero | Kurgan 31, burial 2 | Tibia | ? |
| 28 | Tg62(P) | Podgornoe ozero | Kurgan 29, burial 2 | Tibia | ? |
| 29 | Tg66(Bidj) | Ulug-Kyuzyur | Kurgan 2, burial 1 | Femur | Adult |
| 30 | Tg69(P) | Podgornoe ozero | Kurgan 31, burial 1 | Femur | ? |
| 31 | Tg70(P) | Podgornoe ozero | Kurgan 36, burial 1 | Femur | Adult |
| 32 | Tg71(P) | Podgornoe ozero | Kurgan 35, burial 3 | Tibia | Child |
| 33 | Tg73(P) | Podgornoe ozero | Kurgan 29, burial 3 | Femur | Adult |
| 34 | Tg74(P) | Kichik-Kyuzyur | Kurgan 2, burial 7 | Tibia | Child |
| 35 | Tg76(P) | Kichik-Kyuzyur | Kurgan 3, burial 2 | Tibia | ? |
| 36 | Tg77(P) | Kichik-Kyuzyur | Kurgan 2, burial 4, Skeleton 1 | Humerus | ? |
| 37 | Tg80(P) | Kichik-Kyuzyur | Kurgan 2, burial 5 | Femur | ? |
| 38 | Tg83(P) | Kichik-Kyuzyur | Kurgan 5, burial 4 | Tibia | ? |
| 39 | Tg94(P) | Kichik-Kyuzyur | Kurgan 2, burial 7 | Tibia | ? |
| 40 | Tg97(P) | Kichik-Kyuzyur | Kurgan 6, burial 4 | Femur | ? |
| 41 | Tg99(P) | Kichik-Kyuzyur | Kurgan 5, burial 2 | Tibia | ? |
| 42 | Tg106(P) | Podgornoe ozero | Kurgan 37, burial 1 | Humerus | ? |
| 43 | Tg108(P) | Perevozinskij | Kurgan 10, burial 2 | Tibia | ? |
| 44 | Tg116(P) | Kichik-Kyuzyur | Kurgan 2, burial 4, Skeleton 2 | Humerus | ? |
| 45 | Tg120(P) | Podgornoe ozero | Kurgan 31, burial 1 | Femur | ? |
| 46 | Tg123(P) | Perevozinskij | Kurgan 10, burial 1 | Tibia | ? |

*Stage of Tagar culture: Early Tagar: (Bain) – Bainovo gropu (pre-Podgornovo), (P) – Podgornovo group, (Bidj) – Bidjinski group (post-Podgornovo) Middle Tagar: (S) – Saragash group; Late Tagar: (T) – Tes group.

**Samples from the Middle Stage of Tagar Culture**

| N | Laboratory Code (Tagar Group*) | Burial ground | Description | Bone fragment | Age of Individual |
| --- | --- | --- | --- | --- | --- |
| 1 | Tg2(S) | Tepsej-8 | Kurgan 10a, main burial, Skeleton 1 | Femur | Adult |
| 2 | Tg3(S) | Tepsej-8 | Kurgan 10a, main burial, Skeleton 2 | Femur | Adult |
| 3 | Tg34(S) | Barsuchiha-7 | Kurgan 1, burial 1, Skeleton 2 | Femur | ? |
| 4 | Tg37(S) | Barsuchiha-7 | Kurgan 1, burial 1, Skeleton “Ж” | Tibia | ? |
| 5 | Tg67(S) | Ulug-Kyuzyur | Kurgan 1, burial 2, Skeleton 1 | Femur | Adult |
| 6 | Tg72(S) | Ulug-Kyuzyur | Kurgan 4, burial 2, Skeleton 1 | Tibia | Adult |
| 7 | Tg75(S) | Kichik-Kyuzyur | Kurgan 2, burial 1 | Tibia | ? |
| 8 | Tg91(S) | Barsuchiha-7 | Kurgan 1, burial 1, Skeleton G | Tibia | ? |
| 9 | Tg92(S) | Barsuchiha-1 | Kurgan 3, horizon 3, Skeleton 1 | Tibia | ? |
| 10 | Tg93(S) | Barsuchiha-1 | Kurgan 3, horizon 3, Skeleton 2 | Tibia | ? |
| 11 | Tg96(S) | Barsuchiha-1 | Kurgan 6, horizon 1, Skeleton 4 | Femur | ? |
| 12 | Tg100(S) | Barsuchiha-7 | Kurgan 1, burial 2, Skeleton 2 | Tibia | ? |
| 13 | Tg101(S) | Barsuchiha-1 | Kurgan 6, horizon 1, Skeleton 2 | Humerus | ? |
| 14 | Tg102(S) | Kichik-Kyuzyur | Kurgan 4, burial 2, Skeleton 3 | Tibia | ? |
| 15 | Tg109(S) | Barsuchiha-7 | Kurgan 1, burial 1 Skeleton A | Tibia | ? |
| 16 | Tg110(S) | Barsuchiha-7 | Kurgan 1, burial 1 Skeleton B | Tibia | ? |
| 17 | Tg111(S) | Barsuchiha-7 | Kurgan 1, burial 1 Skeleton C | Tibia | ? |
| 18 | Tg112(S) | Barsuchiha-7 | Kurgan 1, burial 1 Skeleton D | Tibia | ? |
| 19 | Tg113(S) | Barsuchiha-7 | Kurgan 1, burial 1 Skeleton E | Tibia | ? |
| 20 | Tg115(S) | Barsuchiha-7 | Kurgan 1, burial 1 Skeleton F | Tibia | ? |
| 21 | Tg117(S) | Kichik-Kyuzyur | Kurgan 4, burial 5 | Femur | Child |
| 22 | Tg119(S) | Barsuchiha-1 | Kurgan 3, horizon 2 | Tibia | ? |
| 23 | Tg121(S) | Barsuchiha-6 | Kurgan 7, burial 6 | Femur | ? |
| 24 | Tg122(S) | Barsuchiha-6 | Kurgan 7, burial 3, Skeleton 4 | Tibia | ? |

*Stage of Tagar culture: Early Tagar: (Bain) – Bainovo gropu (pre-Podgornovo), (P) – Podgornovo group, (Bidj) – Bidjinski group (post-Podgornovo) Middle Tagar: (S) – Saragash group; Late Tagar: (T) – Tes group.

**Samples from the Late Stage of Tagar Culture**

| N | Laboratory Code (Tagar Group*) | Burial ground | Description | Bone fragment | Age of Individual |
| --- | --- | --- | --- | --- | --- |
| 1 | Tg6(T) | Tepsej-3 | Burial 24A, Skeleton A | Tibia | Adult |
| 2 | Tg9(T) | Tepsej-3 | Burial 24B | Humerus | ? |
| 3 | Tg10(T) | Tepsej-3 | Burial 24A, Skeleton B | Femur | Adult |
| 4 | Tg84(T) | Dolgij Kurgan | Kurgan 4, burial 3 | Tibia | ? |
| 5 | Tg85(T) | Barsuchiha-1 | Kurgan 1, burial 1, Skeleton A | Tibia | ? |
| 6 | Tg86(T) | Barsuchiha-1 | Kurgan 1, burial 1, Skeleton B | Tibia | ? |
| 7 | Tg87(T) | Barsuchiha-1 | Kurgan 1, burial 1, Skeleton C | Tibia | ? |
| 8 | Tg88(T) | Barsuchiha-1 | Kurgan 1, burial 1, Skeleton D | Tibia | ? |
| 9 | Tg89(T) | Barsuchiha-1 | Kurgan 1, burial 1, Skeleton E | Tibia | ? |

*Stage of Tagar culture: Early Tagar: (Bain) – Bainovo group (pre-Podgornovo), (P) – Podgornovo group, (Bidj) – Bidjinski group (post-Podgornovo) Middle Tagar: (S) – Saragash group; Late Tagar: (T) – Tes` group.
