# Supplementary material for: Maternal genetic features of the Iron Age Tagar population from Southern Siberia (1st millennium BC)
Source: PLoS One. 2018 Sep 20;13(9):e0204062. doi: 10.1371/journal.pone.0204062 (PMC6147448; doi:10.1371/journal.pone.0204062)
Supplement: S2 File — (DOCX) [file pone.0204062.s002.docx]

**S2 File. PCR-primers used for amplification of mtDNA fragments.**

| Fragment (position under analysis) of mtDNA without primers | PCR primers | Reference | Aim of analysis |
| --- | --- | --- | --- |
| 15997-16141 | L15996/H16142 | [13] | HVRI sequencing (1 step PCR) |
| 16118-16232 | L16117/H16233 | [13] | HVRI sequencing (1 step PCR) |
| 16210-16347 | L16209/H16348 | [13] | HVRI sequencing (1 step PCR) |
| 16288-16409 | L16287/H16410 | [13] | HVRI sequencing (1 step PCR) |
| 16047-16400 | L16046/H16401 | [14] | HVRI sequencing (1^st^ round of nested PCR) |
| 16074-16366 | L16073/H16067 | [14] | HVRI sequencing (2^nd^ round of nested PCR) |
| 655-698 | 654U/699L | [18] | Status of position 663 (marker of haplogroup A) |
| 4816-4863 | 4815U/4864L | [18] | Status of position 4833 (marker of haplogroup G) |
| 4872-4939 | 4871U/4940L | [18] | Status of position 4917 (marker of haplogroup T) |
| 5164-5178 | 5163U/5179L | [18] | Status of position 5178 (marker of haplogroup D) |
| 6337-6402 | 6336U/6403L | [18] | Status of position 6392 (marker of haplogroup F) |
| 6976-7045 | 6975U/7046L | [18] | Status of position 7028 (marker of haplogroup H) |
| 10001-10047 | 10000U/10048L | [18] | Status of position 10034 (marker of haplogroup I) |
| 10388-10450 | 10387U/10451L | [18] | Status of positions 10398, 10400 (markers of macrohaplogroups M, N) |
| 12304-12351 | 12303U/12352L | [18] | Status of position 12308 (marker of haplogroup U) |
| 13232-13274 | 13231U/13275L | [18] | Status of position 13263 (marker of haplogroup C) |
| 14718-14783 | 14717U/14784L | [18] | Status of position 14766 (marker of haplogroup HV) |
