# Supplementary material for: Maternal genetic features of the Iron Age Tagar population from Southern Siberia (1st millennium BC)
Source: PLoS One. 2018 Sep 20;13(9):e0204062. doi: 10.1371/journal.pone.0204062 (PMC6147448; doi:10.1371/journal.pone.0204062)
Supplement: S3 File — (DOCX) [file pone.0204062.s003.docx]

**S3 File. Previously published data on mtDNA structure from Tagar specimens.** Samples, which shared mtDNA HVRI haplotype with our Tagar series, shown in BOLD.

**Keyser et al., 2009:**

| Specimen number | Code | Description | HVR I haplotype | Haplogroup |
| --- | --- | --- | --- | --- |
| **1** | **S21** | **Anach village, kurgan I, burial 3** | **16126C-16189C-16292T-16292T** | **T** |
| **2** | **S22** | **Anach village, kurgan II, burial 4** | **16126C-16189C-16292T-16292T** | **T** |
| **3** | **S23** | **Tchernogorsk, burial 1** | **16126C-16189C-16292T-16292T-16296T** | **T** |
| **4** | **S24** | **Tchernogorsk, burial 6** | **16129A-16223T-16304C-16391A** | **I** |
| **5** | **S25** | **Oust-Abakansty** | **16093C-16223T—16227G-16278T-16362C** | **G2a** |
| 6 | S26 | Beysky region, burial 3 | 16148T-16223T-6234T-16288C-16298C-16327T | C5 |
| **7** | **S27** | **Bogradsky region, kurgan 133, burial 3** | **CRS** | **U** |
| **8** | **S28** | **Bogradsky region, kurgan 133, burial 3** | **16172C-16179T-16183C-16189C-16232A-16249C-16304C-16311C** | **F1b** |
| **9** | **S29** | **Bogradsky region, kurgan 133, burial 3** | **CRS** | **U** |
| 10 | S32 | Bogradsy region, Abakano-Perevoz II, burial 1 | 16304T-16319A | H |

**Unterlander et al., 2017:**

| Specimen number | Description | HVR I haplotype | Haplogroup |
| --- | --- | --- | --- |
| **1** | **Barsuchij Log, grave 1, individual 1** | **16093C-16223T-16288C-16298C-16327T** | **C5** |
| **2** | **Barsuchij Log, grave 1, individual 2** | **16256T-16270T-16309G** | **U5a1** |
| **3** | **Barsuchij Log, grave 1, individual 3** | **16093C-16223T-16288C-16298C-16327T** | **C5** |
| **4** | **Barsuchij Log, grave 1, individual 4** | **16093C-16223T-16288C-16298C-16327T** | **C5** |
| 5 | Barsuchij Log, grave 1, individual 5 | **16051C-16092C-16129C-16183C-16189C-16362C** | **U2e** |
| **6** | **Barsuchij Log, grave 2, individual 1** | **16189C-16223T-16290T-16319A-16362C** | **A4** |
