# Supplementary material for: Maternal genetic features of the Iron Age Tagar population from Southern Siberia (1st millennium BC)
Source: PLoS One. 2018 Sep 20;13(9):e0204062. doi: 10.1371/journal.pone.0204062 (PMC6147448; doi:10.1371/journal.pone.0204062)
Supplement: S4 File — (DOCX) [file pone.0204062.s004.docx]

**S4 File. Characteristics of the main stages in the progression of the Tagar culture.**

The overall date for Tagar culture existence in the Minusinsk basin – IX – I centuries BC (some authors argue for X BC – I AD chronological frame). Therefore, Tagar culture chronologically cover pre-Scythian, Scythian and Early Xiongnu-Sarmatian times. Tagar culture in the Minusinsk basin succeeded the Karasuk culture of Late Bronze Age.

There are several alternative versions of dividing the Tagar culture in cultural (and chronological stages). The main approach – to distinguish three main stages: Early, Middle and Late Tagar.

One of the main researchers of Tagar culture – Gryaznov M.P., developed a detailed classification of the stages, based on the specificities of the material culture (archaeological data). He distinguished four main Tagar stages (types) and, in addition, the transitional Tagar stages (types) between them. Subsequently, this classification was considered too fractional. However, since we analyze the materials from this author's excavations, we will quote the correspondence between our stages (Early, Middle and Late) and the Tagar cultural types of M.P. Gryaznov.

Funerary practice of overall Tagar culture. The main feature is that the “dead were buried in square of rectangular enclosures made of vertically standing stone slabs, covered with a pyramidal burial mound”. “A general trend in the Tagar burial tradition was that the enclosures and the graves they contained increased in size and depth over time. The stone cists were gradually replaced with timber frames that had strong multi-layered floors. The number of bodies buried in each cist also increases through time” (cited from [Bokovenko et al., 2006]).

**Early Tagar stage:**

Chronology – IX-VI centuries BP (of the end of X-VI). Includes Bainovo (IX-VIII centuries, earliest Tagar sites) and Podgornovo main types and Bidjinski subtype (Late Podgornovo) according to Gryaznov`s classification.

Bainovo type Tagar sites are rare. Enclosure is small, with walls constructed of stone slabs (like in Karasuk burials). Each enclosure contains 1 burial with 1 buried individual.

Podgornovo type: enclosures small and frequently attached to each other, with 1-2 tombs in their center. Timber chamber (rather) or stone cist in the tomb. Single burial are common (but there are rare burials of 2-3 individuals).

**Middle Tagar stage:**

Chronology – VI-III centuries BC. Includes mainly Saragash type according to M.P. Gryaznov classification. Construction of the burial superstructures. Enclosures varies in size (2-300 m^2^) with two or more collective graves (each contains up to 200 buried individuals buried in succession through a special entrance). Complex social stratification appeared (and reflected in burial rites – existence of “elite” and “ordinary” burial sites).

Archaeological data suggests a possible cultural influences from synchronous cultures of the Altay Mountains [Savinov, 2011].

**Late Tagar stage:**

Chronology – II-I centuries BC (up to beginning of I centuries AD). Includes mainly Tes` type Tagar sites from the M.P. Gryaznov classification. There are to types of Tes` burials: 1. Huge kurgans with monumental enclosures build of stone, with one large tomb contains up to several dozens buried individuals; 2. Small ground burials without kurgans.

Archaeological data suggests cultural influences from eastern early nomadic groups (Xiongnu-like and related) [Savinov, 2011].

Some archaeologists propose to allocate Tes` type in a Tes` culture, separated from Tagar culture [Kuzmin, 2011]. But other archaeologist believed, that Tagar culture stages developed on indigenous cultural base and internal influences from culturally contrasting groups had small value on Tagar culture development.
