# Supplementary material for: Maternal genetic features of the Iron Age Tagar population from Southern Siberia (1st millennium BC)
Source: PLoS One. 2018 Sep 20;13(9):e0204062. doi: 10.1371/journal.pone.0204062 (PMC6147448; doi:10.1371/journal.pone.0204062)
Supplement: S5 File — (DOCX) [file pone.0204062.s005.docx]

**S5 File. Frequency of mtDNA haplogroup C4a2a* and C4a2a1 in some modern populations of Siberia and adjacent regions of Eurasia.**

Lineages with A16171G transition belongs to C4a2a*, lineages with A16171G transition belongs to C4a2a1. Clusters C4a2a* and C4a2a1 together formed C4a2a haplogroup.

| Population | Total mtDNA sample number from the population | Reference | Number of C4a2a carriers | Number of C4a2a* carriers | Number of C4a2a1 carriers |
| --- | --- | --- | --- | --- | --- |
| **Southern Siberia and Central Asia, Turkic-speaking groups** | | | | | |
| Altaians | 110 | Derenko et al., 2003 | 4 | - | 4 |
| Altaians-Kizhi | 90 | Derenko et al., 2007 | 5 | - | 5 |
| Telenghits | 71 | Derenko et al., 2007 | 1 | - | 1 |
| Teleuts | 53 | Derenko et al., 2007 | 1 | - | 1 |
| Todjins | 48 | Derenko et al., 2003 | 3 | - | 3 |
| Tofalars | 58 | Derenko et al., 2003 | 5 | 1 | 4 |
| Tofalars | 46 | Starikovskaya et al., 2005 | 6 | - | 6 |
| Tubalars | 72 | Starikovskaya et al., 2005 | 1 | 1 | - |
| Tuvinians | 105 | Derenko et al., 2007 | 6 | - | 6 |
| Tuvinians | 90 | Derenko et al., 2003 | 3 | - | 3 |
| Tuvinians | 95 | Starikovskaya et al., 2005 | 8 | 4 | 4 |
| Shors | 82 | Derenko et al., 2007 | 6 | 1 | 5 |
| Khakassians | 57 | Derenko et al., 2007 | 6 | - | 6 |
| Khakassians | 53 | Derenko et al., 2003 | 5 | - | 5 |
| Kazakhs | 52 | Comas et al., 1998 | 1 | - | 1 |
| **Southern Siberia and Central Asia, Mongolian-speaking groups** | | | | | |
| Buryats | 295 | Derenko et al., 2007 | 7 | - | 7 |
| Buryats | 25 | Starikovskaya et al., 2005 | 2 | - | 2 |
| Barghuts | 149 | Derenko et al., 2012 | 3 | 1 | 2 |
| Khamnigans | 99 | Derenko et al., 2007 | 4 | - | 4 |
| Kalmyks | 110 | Derenko et al., 2007 | 1 | - | 1 |
| Mongols | 47 | Derenko et al., 2007 | 1 | - | 1 |
| Mongols | 103 | Kolman et al., 1996 | 2 | 0 | 2 |
| **Central Siberian populations** | | | | | |
| East Evenks | 45 | Derenko et al., 2007 | 10 | - | 10 |
| West Evenks | 73 | Derenko et al., 2007 | 5 | - | 5 |
| Evenks | 71 | Starikovskaya et al., 2005 | 9 | 2 | 7 |
| Yakuts | 36 | Derenko et al., 2007 | 9 | - | 9 |
| **West Siberian populations** | | | | | |
| Mansi | 98 | Derbeneva et al., 2002 | 4 | - | 4 |
| Kets | 39 | Dernebeva et al., 2002 | 5 | - | 5 |
| Nganasans | 24 | Dernebeva et al., 2002 | 1 | - | 1 |
| **Volgo-Ural Region** | | | | | |
| Bashkirs | 221 | Bermisheva et al., 2002 | 4 | 3 | 1 |

Lineages in the C4a2a (and C4a2a1) cluster represent a significant portion of the haplogroup C diversity in our Tagar series (three out of five lineages). Interestingly, these lineages were not detected in other populations of the Early Iron Age (Scythian and Xiongnu-Sarmatian times) in Central Asia, both in the territory of the Altai-Sayan mountain system and beyond. In particular, C4a2a has never been detected in other (in addition to the Tagar) populations of the Scythian World, such as Pazyryk and Aldy-Bel populations in Siberia and Classic Scythians from the North Pontic steppes. Among the early nomads of the Hunnish time, only a single mtDNA line belonging to the related cluster C4a2 was detected in the Mongolian Xiongnu [33]. Thus, in the Early Iron Age, the mtDNA cluster C4a2a (including C4a2a1) did not appear to be widely distributed in Southern Siberia and Central Asia. This differs substantially from modern populations; the C4a2a lineages (including mainly C4a2a1) are represented at a high frequency in the majority of modern indigenous populations in Southern Siberia (including the modern population of the Minusinsk basin–Khakassians) as well as populations in the adjacent areas of Central Asia and northern areas of Siberia (Supplementary file S11). Thus, they are now a typical component of the mtDNA pool of the modern Turkic-speaking and Mongolian-speaking populations of Central Asia (including Southern Siberia). Therefore, a major increase in the role of this lineage in the genetic composition of Central Asian populations occurred over the past 2000 years. Descendants of the Tagar population (or genetically related populations) could potentially play a substantial role in this process, although this hypothesis requires further research, including analyses of diachronic materials. It should be noted that the initial source of cluster C4a2a1 (and C4a2a1a) appears to be located in Northern China, where variant C4a2a1a was detected in a Neolithic population (at least 5000 years old) [32]. Interestingly, in the mtDNA pool of more recent populations of Northern China (up to the present) and populations of other regions of China, this mtDNA cluster is not represented or is rare.
