# Supplementary material for: Maternal genetic features of the Iron Age Tagar population from Southern Siberia (1st millennium BC)
Source: PLoS One. 2018 Sep 20;13(9):e0204062. doi: 10.1371/journal.pone.0204062 (PMC6147448; doi:10.1371/journal.pone.0204062)
Supplement: S6 File — (DOCX) [file pone.0204062.s006.docx]

**S6 File. Distribution of mtDNA A8 haplogroup lineages in modern and ancient human populations of Eurasia.**

| **Haplotype**  **number** | **MtDNA HVR I haplotype structure** | **Number of the haplotype carriers (whole sample)** | **Population [Reference]** |
| --- | --- | --- | --- |
| 1 | 16223T-16242T-16290T-16319A | 1 (41) | Kurds (Turkmenistan) [Quintana-Murci et al., 2004] |
|  |  | 1 (154) | Dolgans [Fedorova et al., 2013] |
|  |  | 1 (89) | Mongols [Gokcumen et al., 2009] |
|  |  | 1 (206) | Nogays [Bermisheva et al., 2004] |
|  |  | 1 (41) | Turkmens [Quintana-Murci et al., 2004] |
|  |  | 1 (35) | Turkmens [Marchi et al., 2017] |
|  |  | 4 () | Selkups [Tamm et al., 2007] |
|  |  | 1 () | Transylvania [Egyed et al. 2007] |
|  |  | 1 (191) | Yakuts [Fedorova et al., 2003] |
|  |  | **1 (15)** | **Okunevo culture (Bronze Age) [Allentoft et al., 2015; Hollard et al 2018]** |
|  |  | **1 (15)** | **Aldy Bel culture (Iron Age) [Unterlander et al., 2017]** |
| 2 | 16066G-16223T-16242T-16290T-16319A | 1 (295) | Buryats [Derenko et al., 2007] |
|  |  | 1 (149) | Barghuts [Derenko et al., 2012] |
|  |  | 1 (154) | Dolgans [Fedorova et al., 2013] |
|  |  | 2 (76) | Mongols [Marchi et al., 2017] |
|  |  | 3 (176) | Yakuts [Pakendorf et al., 2006] |
|  |  | 1 (206) | Nogays [Bermisheva et al., 2004] |
|  |  | 2 (36) | Uzbeks [Martinez-Cruz et al., 2011] |
| 3 | 16066G-16093C-16223T-16242T-16290T-16319A | 1 (110) | Kalmyks [Derenko et al., 2007] |
| **4** | **16075C-16223T-16242T-16290T-16319A** | **1 (79)** | **Tagar culture [This work]** |
| 5 | 16147A-16223T-16242T-16290T-16319A | 1 (237) | Altaian Kazakhs [Gokcumen et al., 2009] |
| 6 | 16242T-16290T-16293G-16319A | 1 (95) | Tuvinians [Starikovskaya et al., 2005] |
| **7** | **16223T-16242T-16278T-16290T-16319A** | **1 (79)** | **Tagar culture [This work]** |
|  |  | **1 (71)** | **Pazyryk culture [Unterlander et al., 2017]** |
|  |  | 1 (52) | Kazakhs (Kazakhstan) [Comas et al., 1998] |
|  |  | 1 (43) | Uzbeks [Quintana-Murci et al., 2004] |
| **8** | **16129A-16223T-16242T-16278T-16290T-16319A** | **3 (79)** | **Tagar culture [This work]** |
| **9** | **16193T-16223T-16242T-16278T-16290T-16319A** | **1 (79)** | **Tagar culture [This work]** |
| **10** | **16223T-16242T-16278T-16290T-16311C-16319A** | **2 (79)** | **Tagar culture [This work]** |
| **11** | **16093C-16223T-16242T-16278T-16290T-16311C-16319A** | **1 (71)** | **Pazyryk culture [Unterlander et al., 2017]** |

Unlike C4a2a, the role of A8 in the gene pool of the population of Southern Siberia has reduced substantially over the past ~2000 years. This cluster was widely represented in Scythian-Siberian groups, including the Tagar, Pazyryk and Aldy Bel culture populations [4; this study]. In the Tagar population, most lineages of haplogroup A are represented by the A8 subcluster, and this population shows the highest frequency of A8 reported (greater than 10%) (Fig 8; Supplementary file S12). But haplogroup A8 was absent in other Iron Age populations. Among the other ancient groups A8 haplogroup lineages have only been identified in the Bronze Age Okunevo [Hollard et al., 2018] and Karasuk (our data, manuscript in preparation) culture populations, which is the population that preceded the Tagar culture in the region.

Haplogroup A8 is a rare component of the gene pool of modern Eurasian populations and is mainly distributed in Eastern (including the south of Siberia) and Western Central Asia (Fig 8; Supplementary file S12). In modern populations of Western Eurasia, it is even more rare [62]. Interestingly, almost all variants of A8 found in the ancient Scythian-Siberian populations (except for one variant found in the Tagar group) are characterized by a common haplotype with a C16278T substitution and obviously belong to a subcluster of haplogroup A8 that has not been annotated to date. The variant lacking С16278T is dominant in modern populations. Rare carriers of a single lineage of A8 with C16278T have been detected in a few populations in Western Central Asia, i.e., in Kazakhs [46] and Uzbeks [52] (Fig 8; Supplementary file S12). In the territory of South Siberia and adjacent areas of Eastern Central Asia, A8 lineages with C16278T have not been detected.
