# Supplementary material for: Maternal genetic features of the Iron Age Tagar population from Southern Siberia (1st millennium BC)
Source: PLoS One. 2018 Sep 20;13(9):e0204062. doi: 10.1371/journal.pone.0204062 (PMC6147448; doi:10.1371/journal.pone.0204062)
Supplement: S7 File — (DOCX) [file pone.0204062.s007.docx]

**S7 File. Autosomal STR-loci allelic profiles and results of sex determination of the Tagar individuals.**

| Sample | Sex | D3S1358 | vWA | FGA | D8S1179 | D21S11 | D18S51 | D5S818 | D13S317 | D7S820 |
| --- | --- | --- | --- | --- | --- | --- | --- | --- | --- | --- |
| Tg14 | XY | 14/15 | 17/18 | 21/23 | 10/14 | 30/30* | - | 11/12 | 10/12 | 11/11* |
| Tg18 | XY | 15/15 | 15/19 | 24/24* | 13/13 | 28/29 | 14/14* | 9/11 | 10/14 | 11/12 |
| Tg26 | XY | 14/16 | 14/15 | 23/25 | 10/14 | 30/32.2 | 13/14 | 11/12 | 12/13 | 11/12 |
| Tg66 | XY | 14/17 | 15/16 | 24/25 | 12/13 | 29/31 | 14/19 | 10/12 | 8/12 | 10/11 |
| Tg70 | XY | 15/16 | 17/17 | 22/23 | 10/14 | 30/31.2 | - | 10/12 | 11/11 | 11/11* |
| Tg119 | XY | 16/17 | 15/18 | 21/21* | 13/13 | 31/31.2 | 14/14* | 12/12 | 12/12* | - |

STR genotyping was performed several times for each individual. In the final version of the results were only alleles stably supported by the analysis of repeated PCR. We are aware that some homozygosity cases may be due to inability to amplify longer allelic variants, especially for STR-loci having a greater length of amplicons (FGA, D8S51, D7S820) (the cases marked by *). Homozygous status is set when the amplification product of the second allele never been identified. Comparison with the corresponding profiles STR-loci laboratory staff used as an additional control to exclude intra-laboratory contamination. “-“ – means “No data”.
